# Somatic Embryogenesis: Identified Factors that Lead to Embryogenic Repression. A Case of Species of the Same Genus

**DOI:** 10.1371/journal.pone.0126414

**Published:** 2015-06-03

**Authors:** Geovanny I. Nic-Can, Rosa M. Galaz-Ávalos, Clelia De-la-Peña, Armando Alcazar-Magaña, Kazimierz Wrobel, Víctor M. Loyola-Vargas

**Affiliations:** 1 Unidad de Bioquímica y Biología Molecular de Plantas, Centro de Investigación Científica de Yucatán, Calle 43 No. 130, Col. Chuburná de Hidalgo, CP 97200, Mérida, Yucatán, México; 2 Unidad de Biotecnología, Centro de Investigación Científica de Yucatán, Calle 43 No. 130, Col. Chuburná de Hidalgo, CP 97200, Mérida, Yucatán, México; 3 Department of Chemistry, University of Guanajuato, L. de Retana 5, CP 36000 Guanajuato, Mexico; Universidad Miguel Hernández de Elche, SPAIN

## Abstract

Somatic embryogenesis is a powerful biotechnological tool for the mass production of economically important cultivars. Due to the cellular totipotency of plants, somatic cells under appropriate conditions are able to develop a complete functional embryo. During the induction of somatic embryogenesis, there are different factors involved in the success or failure of the somatic embryogenesis response. Among these factors, the origin of the explant, the culture medium and the *in vitro* environmental conditions have been the most studied. However, the secretion of molecules into the media has not been fully addressed. We found that the somatic embryogenesis of *Coffea canephora*, a highly direct embryogenic species, is disrupted by the metabolites secreted from *C*. *arabica*, a poorly direct embryogenic species. These metabolites also affect DNA methylation. Our results show that the abundance of two major phenolic compounds, caffeine and chlorogenic acid, are responsible for inhibiting somatic embryogenesis in *C*. *canephora*.

## Introduction

The production of a whole plant from a single somatic cell is a clear confirmation that cells contain the genetic information necessary to develop a new and functional plant [1]. This process is known as somatic embryogenesis (SE), and is a powerful biotechnological process that allows the mass production of economically important cultivars. Furthermore, SE is also an attractive system to study the morphology, biochemistry, genetic and molecular mechanisms of embryo development [2]. This system is built on the concept of cellular totipotency, which is the ability of a single cell to divide and produce a complete and functional plant. A certain subset of cells, under specific induction conditions, can generate embryogenic cells that lead to the initiation of the somatic embryo [1,3].

There are different factors involved in the success or failure of the SE response. Among these factors are the species, the origin of the explant, the culture medium, the type and concentration of the growth regulators used, the nitrogen and carbon sources, and the *in vitro* environmental conditions [4–6]. Another important factor that affects the SE response is the release of organic molecules by the explants into the culture medium. Some of the secreted molecules that have been demonstrated to induce or modulate the SE response [7–9] have been classified as polysaccharides, amino acids, growth regulators, vitamins [10], proteins and lipophilic molecules [11–13]. Other compounds, mostly secondary metabolites, have been found to inhibit the embryogenic response of the cells. For instance, in *Daucus carota*, SE is inhibited by a chemical compound of low molecular mass that is released in response to high density cell cultures [14]. This inhibitory compound was identified as 4-hydroxybenzyl alcohol (4-HBA) [15] and its accumulation in embryogenic cell cultures suppresses the rapid division of cells, mainly at early globular stages, inhibiting SE. Subsequently, it was observed that during embryogenic induction in *Larix leptolepis*, some inhibitory factors present in the medium culture also affected the SE response [16]. These inhibitory molecules were isolated and identified as vanillyl benzyl ether (VBE) [17] and 4-[(phenylmethoxy) methyl] phenol (4-PMP) [18]. VBE causes aberrations, particularly in the suspensor, which arrest the nutrient supply and growth regulator flux, inhibiting the development of somatic embryos in *L*. *leptolepis*. Besides VBE, the presence of 4-PMP in the culture medium strongly suppresses SE [18]. The molecular mechanism by which secondary metabolites affect the SE response is not understood. However, it seems that epigenetic modulation could be involved [19–22].

It has been shown that DNA methylation is important for somatic embryo development [23–26]. For instance, in *Pinus nigra*, the higher levels of DNA methylation correspond to a non-embryogenic line, but low levels promote SE [27]. Similar events have also been reported in other species, such as *Rosa x hybrid* [28] and *Eleuterococcus senticosus* [29]. Furthermore, in both *D*. *carota* and *Cucurbita pepo*, the formation of embryogenic cells is related to an increase in DNA methylation levels [23,24]. It was also found that during the generation of pro-embryogenic mass (Pm), the methylation of DNA decreases, but it gradually reverts in accordance with embryo development [23]. More recently, similar events were observed during the SE of *C*. *canephora* [26], where the maturation of the embryo was marked by an increase in the levels of DNA methylation. In contrast, disturbances in DNA methylation patterns caused by 5-azacytidine (5-AzaC, demethylating agent) cause the loss of embryogenic potential in cultures of *Medicago truncatula* and *D*. *carota* [30,31] as well as in the early stages of *C*. *canephora* SE [26]. On the other hand, it has been shown that there exists a negative relation between phenolic compounds and the DNA methylation levels [32]. Several plant-derived compounds have been proposed as DNA methylation modulators, including polyphenols, alkaloids, organosulfur compounds and terpenoids, which have been found to have important functions [21,22]. However, the role of secreted molecules into the media has not been fully addressed.

*C*. *arabica* and *C*. *canephora* are the most important species of the genus *Coffea*. Some of the distinctive characteristics of these species are that *C*. *canephora* is resistant to some diseases, while *C*. *arabica* produces coffee of high quality. These species combined produce 100% of the commercial coffee around the world. The biology of the species makes them incompatible and unable to produce hybrids. However, plant tissue culture can be used to resolve this issue.

SE of *C*. *arabica* has been explored by several groups around the world [33–36]. The systems used by these groups take a large amount of time to produce somatic embryos. We have developed a SE method that produces viable embryos within a few weeks in *C*. *canephora* [37]. However, under the same induction conditions, *C*. *arabica* is not able to produce somatic embryos.

This background provides evidence that compounds released by somatic cells could determine whether the embryogenic process succeeds or not, somehow affecting DNA methylation. Therefore, the central aim of the present study is to assess whether the secondary metabolites secreted by *C*. *arabica* into the culture medium affect the SE response and DNA methylation in *C*. *canephora*, a highly embryogenic species. We found that the SE of *C*. *canephora* is disrupted by the metabolites secreted from *C*. *arabica*, which also affect DNA methylation.

## Results

### Differential response to somatic embryogenesis in coffee

**Fig 1:**
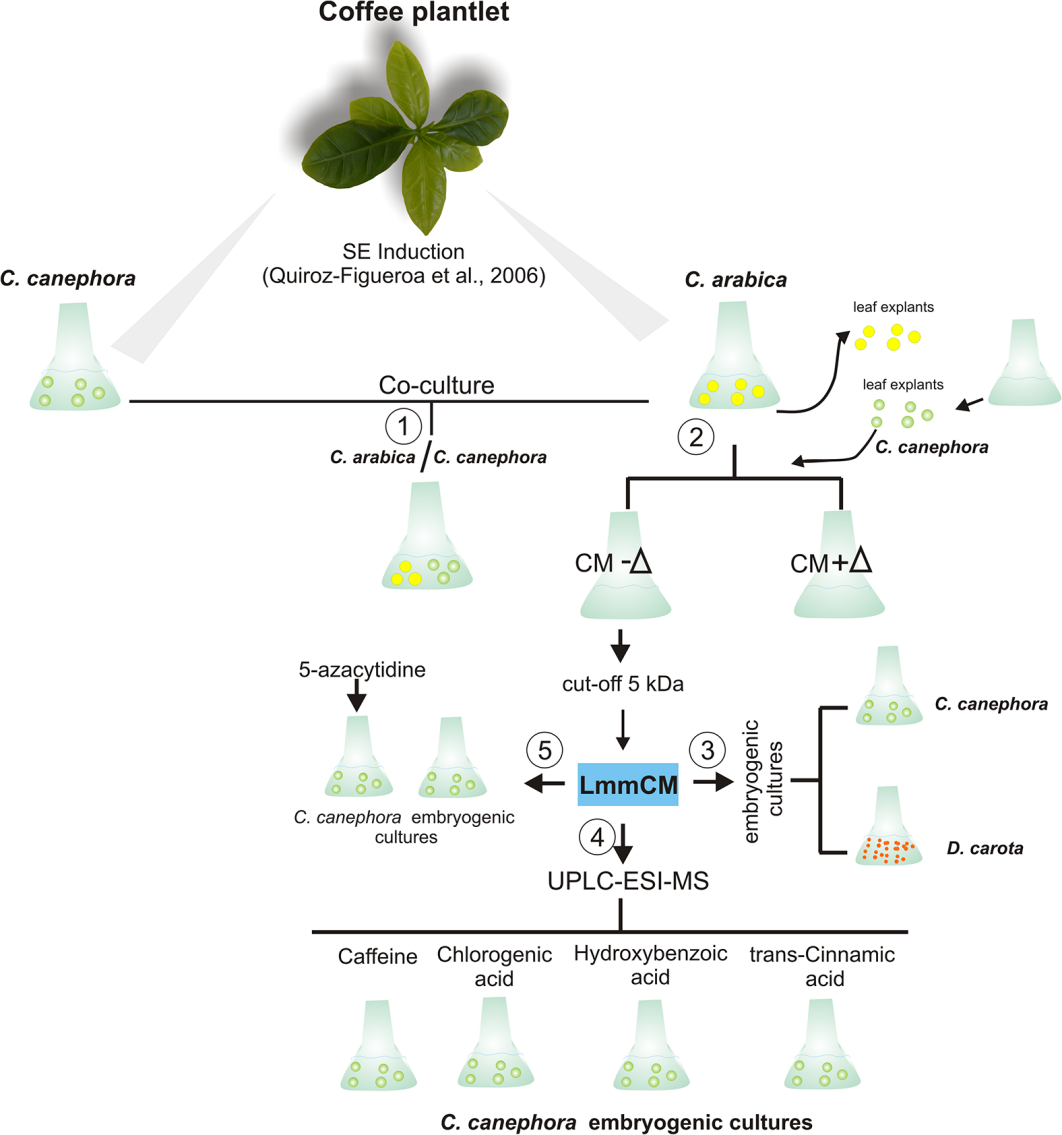
Schematic representation of the experimental procedure used in this study. Leaf explants of *Coffea arabica* (*Ca*) and *C*. *canephora* (*Cc*) were cultivated in the somatic embryogenesis (SE) induction media. In order to evaluate the factors involved in the inhibition of SE in *Ca*, the next steps were followed: 1. *Ca* and *Cc* explants were co-cultured together in the same medium. 2. The explants from *Ca* were discarded and the conditioned medium (CM) was used either fresh (CM-∆) or autoclaved (CM+∆) to culture the *Cc* explants. 3. The CM from seven days was fractionated with a 5 kDa cut-off membrane and the LmmCM obtained was added into the embryogenic cultures of *Cc* and *Daucus carota* (*Dc*). 4. The LmmCM was analyzed by GC-MS or UPLC-ESI-MS and the commercial identified phenolic compounds were added to *Cc* cultures. 5. The effect of LmmCM in DNA methylation was compared with that of the 5-azacytidine. Green circles: *Cc* leaf explants; yellow circles: *Ca* leaf explants; orange circles: *Dc* embryogenic cells.

**Fig 2:**
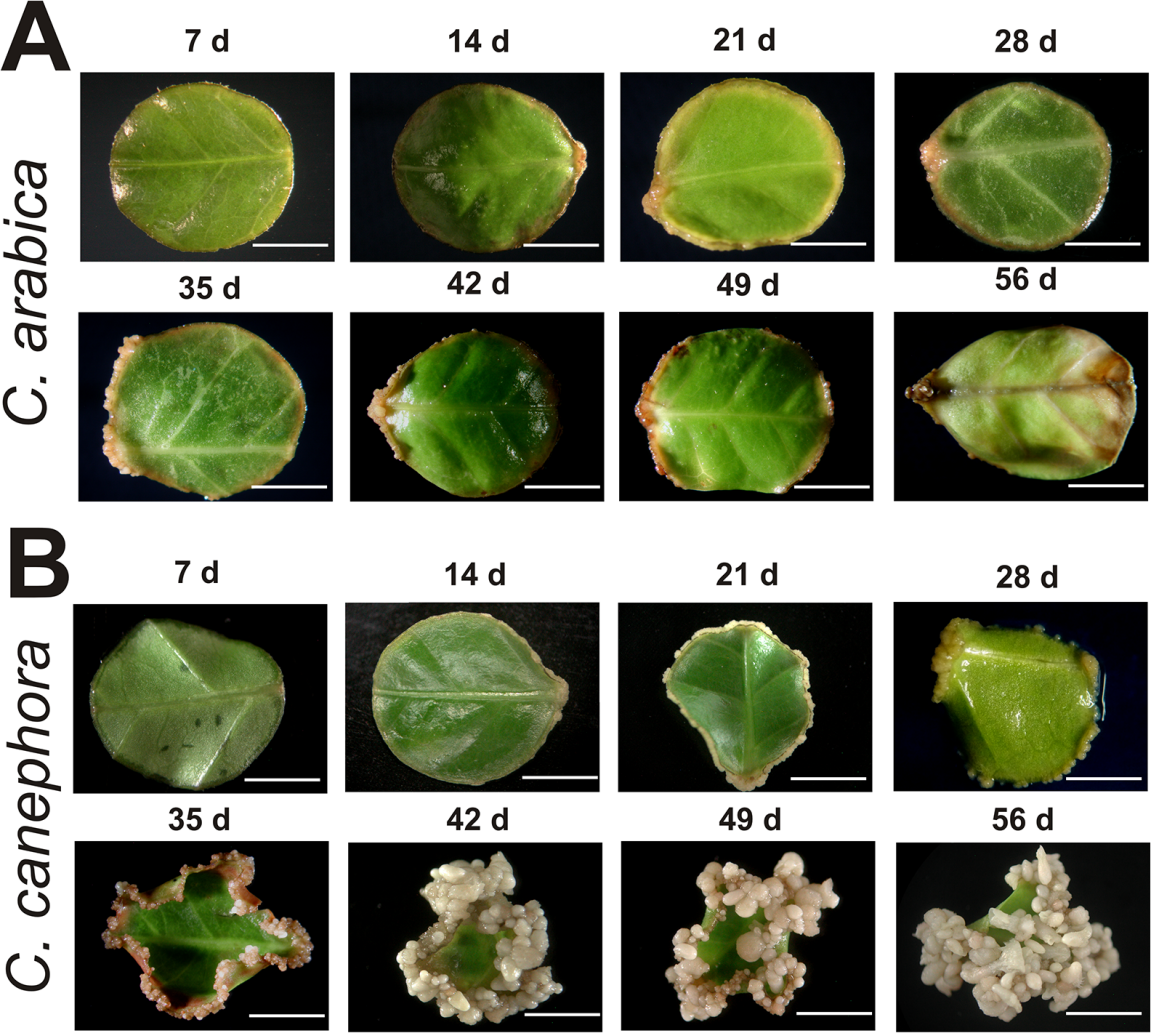
The somatic embryogenesis process in *Coffea arabica* and *C*. *canephora*. Leaf explants were cultured in liquid medium using 5 μM of 6-benzyladenine. A. Leaf explants of *C*. *arabica* during the 56th day of somatic embryogenesis induction. The samples were taken every seven days. B. Leaf explants of *C*. *canephora* during the somatic embryogenesis process. The samples were taken every seven days. Bars = 5 mm.

In the present work, the plantlets from both species were subjected to the same *in vitro* preconditioning treatment with the growth regulators 1-naphthalene acetic acid (NAA) and kinetin (KIN), and then the explants were incubated with 6-benzyladenine (BA) to induce SE (see Material and Methods; Fig 1). After 56 days under these conditions, SE development in both species was compared. In *C*. *arabica* (Fig 2A), the first morphological change observed in the explants, around 14 days after induction (dai), was a small amount of growth of dedifferentiated tissue around the explant, which was most visible between 21 and 28 dai. At 35 dai, an increase in the growth of dedifferentiated tissue was observed, but from day 42 to day 56 dai, cell proliferation stopped and both the callus-like structures and the explants showed a rapid phenolic oxidation and died. However, in the case of *C*. *canephora* (Fig 2B), the SE response was very efficient and the first changes in the explants were observed between 7 and 14 dai, with a thickening in the edge of the explant and the beginning of cell proliferation, which quickly increased between 21 and 28 dai, generating the so-called Pm. The high rate of cell division in Pm allowed the start of the first embryogenic stage, the globular (G), at 35 dai. The development of the next embryogenic structures, heart (H) and torpedo (T), were predominant from 42 to 49 dai and finally, at 56 dai, the cotyledonary (C) embryos were completely developed.

**Fig 3:**
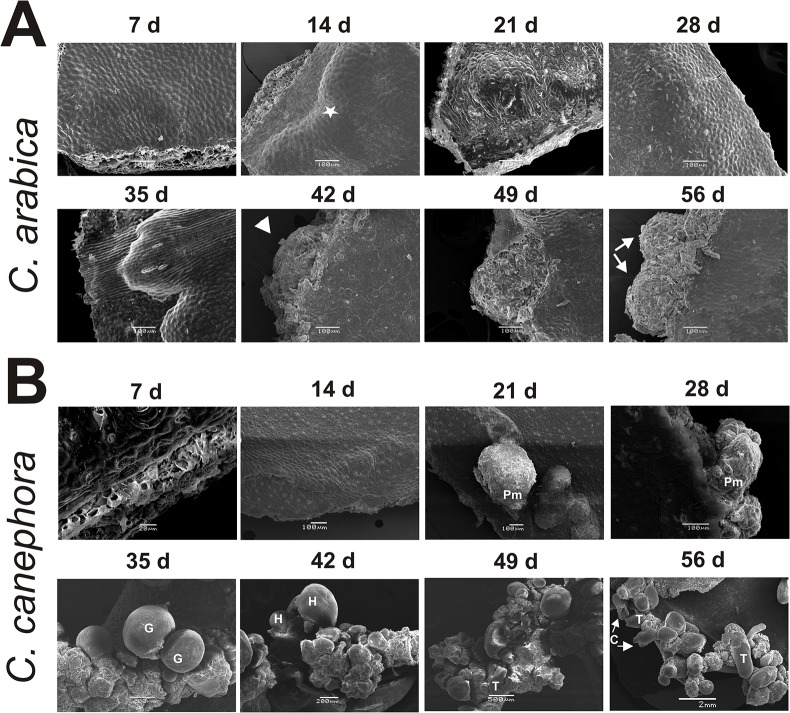
Scanning electron microscopy (SEM) examination of the explants of *Coffea arabica* and *C*. *canephora* under embryogenic conditions. A. SEM of *C*. *arabica* explants during the 56-day somatic embryogenesis process. The white star indicates the cellular growth, and the white arrows show the generation of proembryogenic mass-like structures at 42 days and the development of a globular-like stage with an aberrant surface at 56 days. B. SEM of *C*. *canephora* explants during the somatic embryogenesis process. At 21 and 28 days after induction (dai), the proembryogenic mass (Pm) emerging from the mesophyll region of the explant is shown. The globular stage (G) appears after five weeks, with a well-differentiated protoderm structure. Heart stages (H) can be observed at 42 dai, torpedo stage (T) at 49 dai and cotyledonary stage (C) at 49 and 56 dai.

In order to evaluate SE development in both species more closely, the SE process was observed every week by scanning electron microscopy (SEM) (Fig 3). In the case of *C*. *arabica* (Fig 3A), while cell proliferation starts at seven dai, at 14 days the cellular growth is more evident, and it can be observed that the growth initiates in the wounded cells. Between 28 and 35 dai, the accumulation of cell proliferation begins to break the epidermal cells of the explant, and between 42 to 49 dai, it is possible to observe semi-organized structures, which are more evident at 56 dai. These structures could correspond to early embryogenic stages such as globular-like structures. However, we observed several morphological defects; for instance, the presence of non-embryogenic cells as well as the absence of protoderm (Fig 3A, 56 dai), which is crucial to normal embryo development [8].

On the other hand, when we observed, through SEM, the development of SE in *C*. *canephora* (Fig 3B), we found interesting similarities and important differences in comparison with *C*. *arabica* (Fig 3A). For instance, SE in *C*. *canephora* proceeds similarly to *C*. *arabica* during the first 14 dai. However, cell growth and proliferation in *C*. *canephora* was more active and abundant, allowing the establishment of the Pm between 21 and 28 dai. At day 35 dai in *C*. *canephora*, the cells’ organization leads to the formation of the G stage, which is characterized by its spherical form and the presence of the protoderm. Then, by 42 dai, it can be observed that the embryos previously in the G stage have developed into H embryos, the Pm has increased, and the generation of new G embryos has also increased. During the last two weeks, important changes were observed in the SE of *C*. *canephora*. First, at the H stage, the elongation of the embryo and the development of cotyledonary primordia allow the formation of the T stage, which is the quickest transition into the C embryo stage. Since SE is an asynchronous process, by 56 dai it is possible to observe all the embryogenic stages, and yet it is possible to distinguish the proliferation of new G embryos (Figs 2B and 3B).

### Compounds secreted by *Coffea arabica* explants inhibit SE in other species

**Fig 4:**
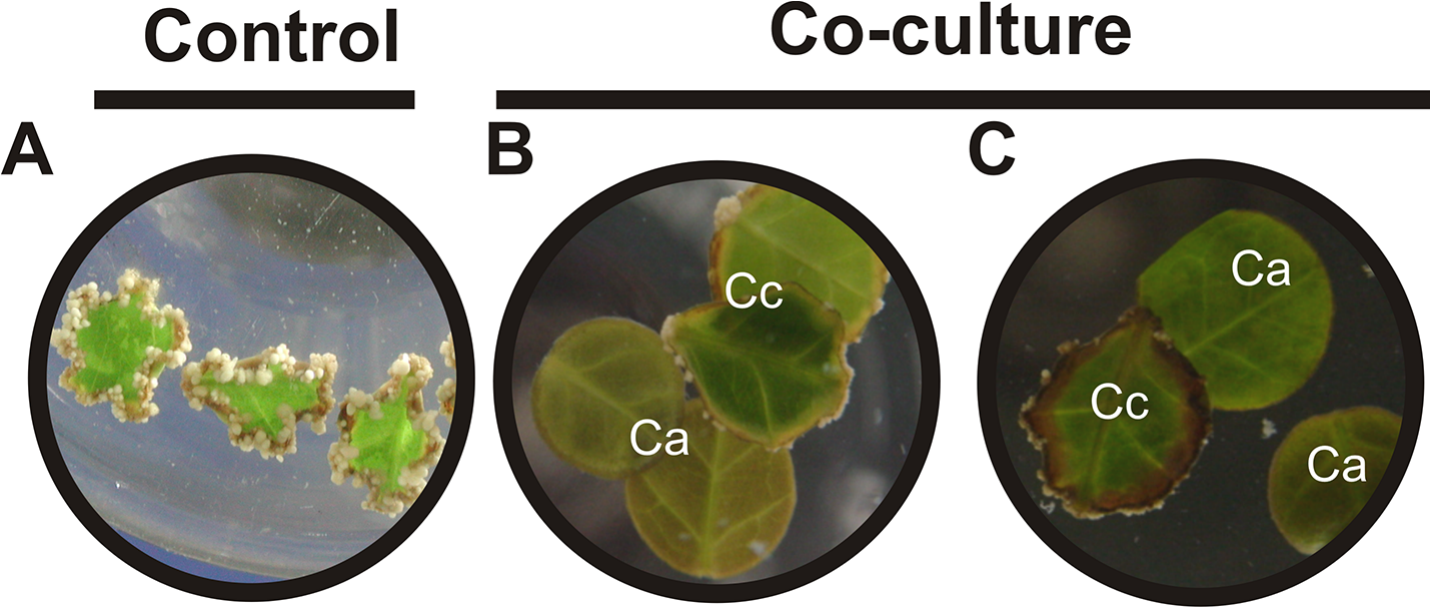
Inhibition assays during the somatic embryogenesis of *C*. *canephora*. A. *C*. *canephora* control after 42 dai with any treatment. Embryogenic explants of *C*. *canephora* (Cc) 21 dai were co-cultured together with 7-day-old explants of *C*. *arabica* (Ca). B. The picture was taken one week later. C. The picture was taken three weeks later.

Previous reports have indicated that one of the reasons why an explant does not respond to *in vitro* culturing is the secretion of inhibitory compounds into the culture medium [38,39]. In this context, we wondered whether the impaired SE in *C*. *arabica* is due to extracellular compounds released into the medium. Therefore, we added 21 dai *C*. *canephora* explants into the medium of 7 dai *C*. *arabica* and co-cultured them together for two weeks (Fig 4B; See Fig 1, step 1). Surprisingly, we found that the co-culture of *C*. *arabica* and *C*. *canephora* negatively affects the development of the SE process in *C*. *canephora*; this negative effect was reflected by a significant reduction in cell proliferation of the Pm and a rapid phenolic oxidation around the explant, which was more evident three weeks after the co-culturing (Fig 4C). This finding strongly suggests that the explants of *C*. *arabica* release inhibitory molecules into the culture medium, impairing even a highly embryogenic process.

**Fig 5:**
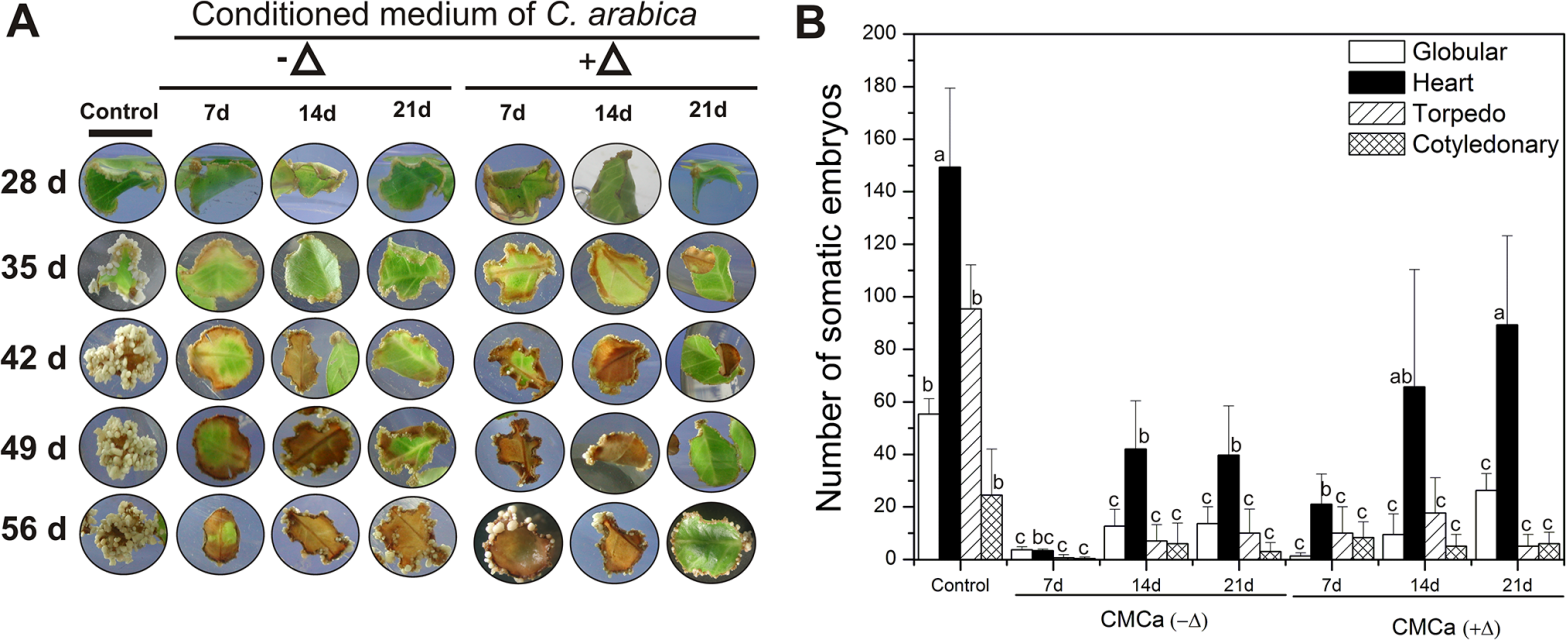
Effect of the conditioned medium of *C*. *arabica* on the formation of somatic embryos in *C*. *canephora*. A. Temporal course of explants from *C*. *canephora* leaves under embryogenic induction without (control) or with the presence of the conditioned medium (CM) of *C*. *arabica* fresh (-∆) or autoclaved (+∆) from 7, 14 and 21 dai, as indicated in Materials and Methods. B. The embryogenic response of *C*. *canephora* evaluated after five weeks without (control) or with the presence of CMCa-∆ and CMCa+∆. Error bars represent the SE (*n* = 3). Bars marked with different letters indicate statistically different values between each embryogenic stage at a given time according to a Tukey test (*P* ≤ 0.01). The experiment was carried out independently three times.

To determine whether the inhibitors in the conditioned medium (CM) of *C*. *arabica* and, therefore, their effects accumulate during the period of culture, we cultured explants of *C*. *canephora* of 21 dai in CM of *C*. *arabica* of 7, 14 and 21 dai without any additional treatment (Fig 5; See Fig 1 step 2). The embryogenic response of *C*. *canephora* was evaluated every seven days (Fig 5A), and the results of the formation of total somatic embryos on the CM were accounted for after 35 days of incubation (Fig 5B). It was found that the CM of *C*. *arabica* at any time point was able to inhibit the embryogenic process in *C*. *canephora*. The CM from seven dai inhibited the SE of *C*. *canephora* more strongly (~93%), while the CM from 14 and 21 days reduced the embryogenic response by 80% in comparison with the control (Fig 5B). Additionally, in order to know whether the inhibitory compounds were heat-resistant, the CM from 7, 14 and 21 dai was autoclaved (+Δ) and used as in the previous experiment. Under this condition, a small increase in the embryogenic response was observed, such as a gradual increase in the number of somatic embryos, depending on the age of the CM used. For instance, when the CM was heated, the amount of *C*. *canephora* embryos in the H stages was higher in comparison with the CM that was not heated, although the embryos at T or C stages did not change in either condition (Fig 5B), indicating that the secreted inhibitory compounds from the CM of *C*. *arabica* are heat-resistant.

Since when we add CM we are also adding inorganic salts, we performed an experiment to rule out the effect of the inorganic components of the Yasuda medium. We extracted the CM with ethyl acetate and used the components in the organic phase to inhibit SE in *D*. *carota* (S1 Fig). The inhibitory effect was the same as that of the CM without the ethyl acetate partition, suggesting that the inhibition was not due to the presence of additional inorganic salts.

**Fig 6:**
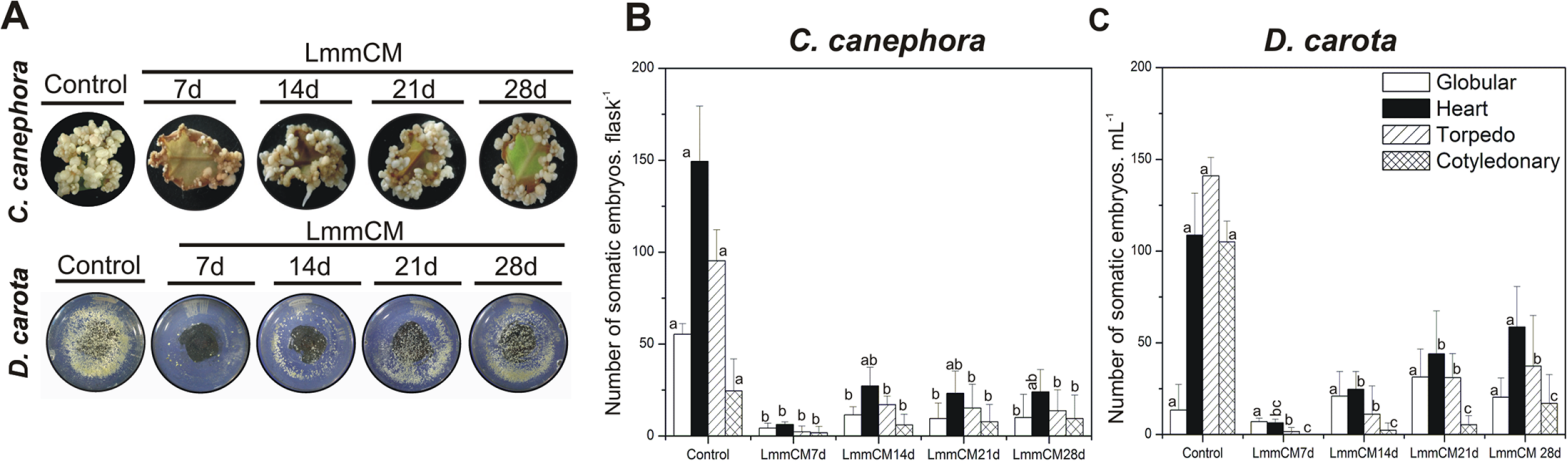
Effect of low molecular mass compounds secreted by explants of *C*. *arabica* on the embryogenic cultures of *C*. *canephora* and *D*. *carota*. A. Morphological effects on the explants of *C*. *canephora* and suspension cells of *D*. *carota* under embryogenic conditions treated with low molecular mass of conditioned medium (LmmCM). B. Fraction of LmmCM of *C*. *arabica* of 7, 14, 21 and 28 dai was added to the embryogenic cultures, and its effect was plotted against the number of somatic embryos generated. C. The LmmCM of 7, 14, 21 and 28 days was added at the beginning of embryogenic induction in *D*. *carota*. The LmmCM of 7, 14, 21 and 28 days was added at 21 days after the embryogenic induction in the embryogenic cultures of *C*. *canephora*. The number of somatic embryos in *C*. *canephora* and *D*. *carota* at different developmental stages was counted at 56 and 14 days, respectively. Controls were cultivated in the absence of LmmCM. Error bars represent the standard error (*n* = 3). Different letters in bars represent the statistical significance of mean differences between each embryogenic stage at a given time according to the Tukey test (*P* ≤ 0.01). The experiment was carried out three times.

It has been reported that chemical factors implicated in the impairment of SE are substances of low molecular weight [14,16]. Therefore, in order to test this theory, the CM of *C*. *arabica* from 7, 14, 21 and 28 dai was separated by molecular mass, through a 5 kDa cut-off membrane (Fig 1, step 3), and the fraction LmmCM was tested in *C*. *canephora* and *D*. *carota* SE (Fig 6). The LmmCM was added separately to the embryogenic culture of 21 dai *C*. *canephora*, and the embryogenic response was evaluated at day 35. It was observed that the presence of LmmCM from 7 dai dramatically reduced the embryonic response, up to 95.5%, whereas the LmmCM from 14, 21 and 28 dai caused an inhibition between 81% and 83%, respectively (Fig 6B). In the case of the embryogenic culture of *D*. *carota*, the LmmCM was added at the beginning of the embryogenic induction, and the embryogenic response was evaluated after 14 days. The embryogenic response in *D*. *carota* was also inhibited (Fig 6A), particularly the LmmCM collected from 7 dai, which strongly stops the development of the embryogenic structures (Fig 6C). These results indicate that the inhibitory molecule(s) present in the CM of *C*. *arabica* can impair the embryogenic development of highly embryogenic cultures such as *C*. *canephora* and *D*. *Carota*.

### Identification of the chemical nature of the secreted compounds by *Coffea arabica* explants

**Fig 7:**
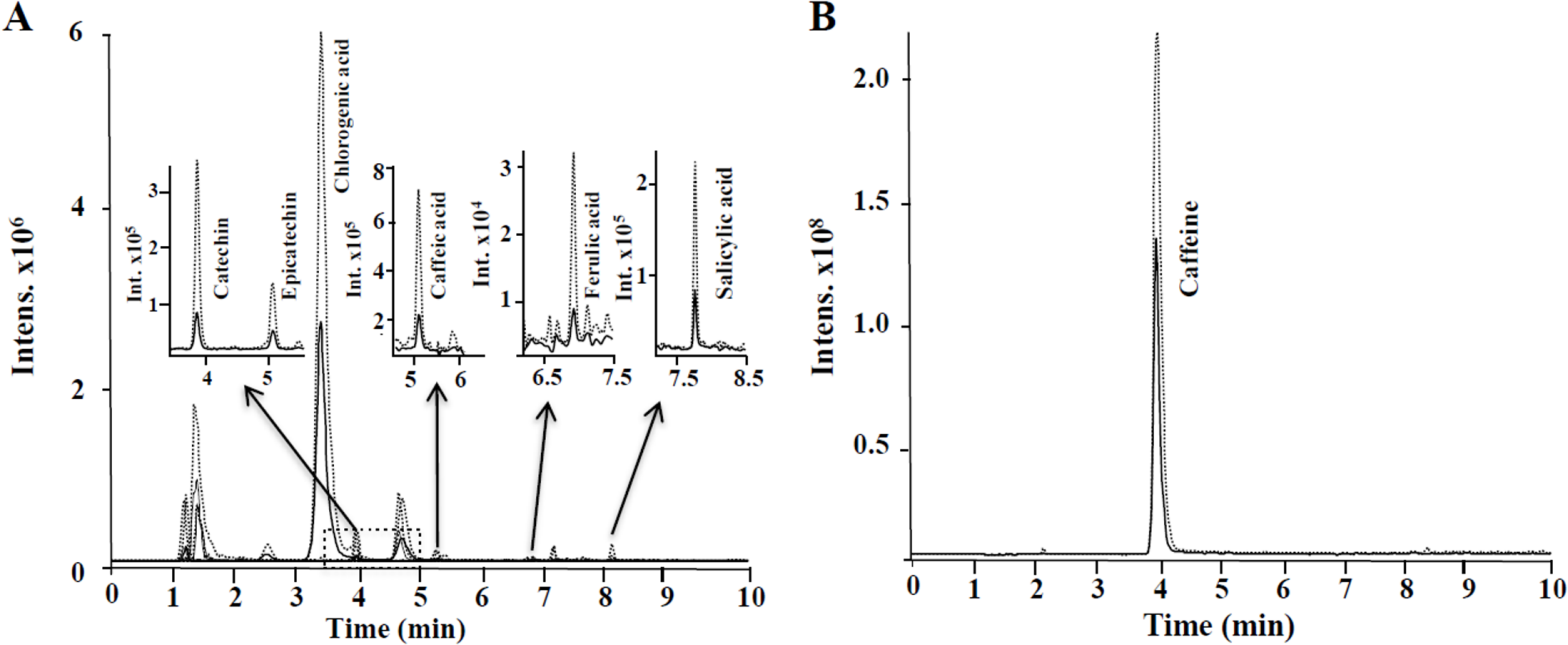
Selected extracted ion chromatograms obtained of LmmCM from *C*. *arabica* by UPLC-ESI-ITMS. A. Phenolic compounds detected in negative electrospray ionization mode. B. Caffeine was detected under positive electrospray ionization. Concentrations of phenolics compound are listed in Table 1.

In order to identify which compounds are present in the LmmCM fraction from seven dai, we analyzed, by GC-MS and UPLC-ESI-ITMS, the CM from *C*. *arabica* as described in Materials and Methods. It was found by GC-MS analysis that the LmmCM is composed of one prominent compound, which was identified as caffeine, whereas hydroxybenzoic acid and trans-cinnamic acid showed lower signal intensity (S2 Fig). The analysis of the LmmCm fraction by UPLC-ESI-ITMS led to the identification of the eight compounds listed in Table 1. As all of the compounds were tentatively identified as phenolics, we included 34 phenolic compounds as standards (S1 Table). The compounds 1–32 were run in negative ionization mode, whereas compounds 27, 33 and 34 were operated in positive ionization mode. Using the standards as a guide, as well as the retention time and extracted ion chromatograms, we detected and quantified eight phenolic compounds (Fig 7) from two independent experiments. Among the identified compounds, the benzoic, ferulic, salicylic and caffeic acids, as well as catequine and epicatequine, were present in low concentrations in the LmmCM, whereas chlorogenic acid and caffeine represent 98% of the phenolic compounds identified (Table 1).

**Table 1:** Concentration of small phenolic compounds identified in two independent samples of LmmCM from *C*. *arabica* by UPLC-ESI-ITMS.

Phenolic compound	LmmCM_1_ (μmoles.flask^-1^)	LmmCM_2_ (μmoles.flask^-1^)
**Benzoic acid**	0.26	0.39
**Caffeine**	11.042	10.982
**Caffeic acid**	0.017	0.039
**Catequine**	0.034	0.087
**Chlorogenic acid**	1.715	1.986
**Epicatequine**	0.029	0.050
**Ferulic acid**	0.0178	0.024
**Salicylic acid**	0.186	0.275

### Effect of caffeine, chlorogenic, hydroxybenzoic and trans-cinnamic acids on the formation of somatic embryos on *C*. *canephora*

**Fig 8:**
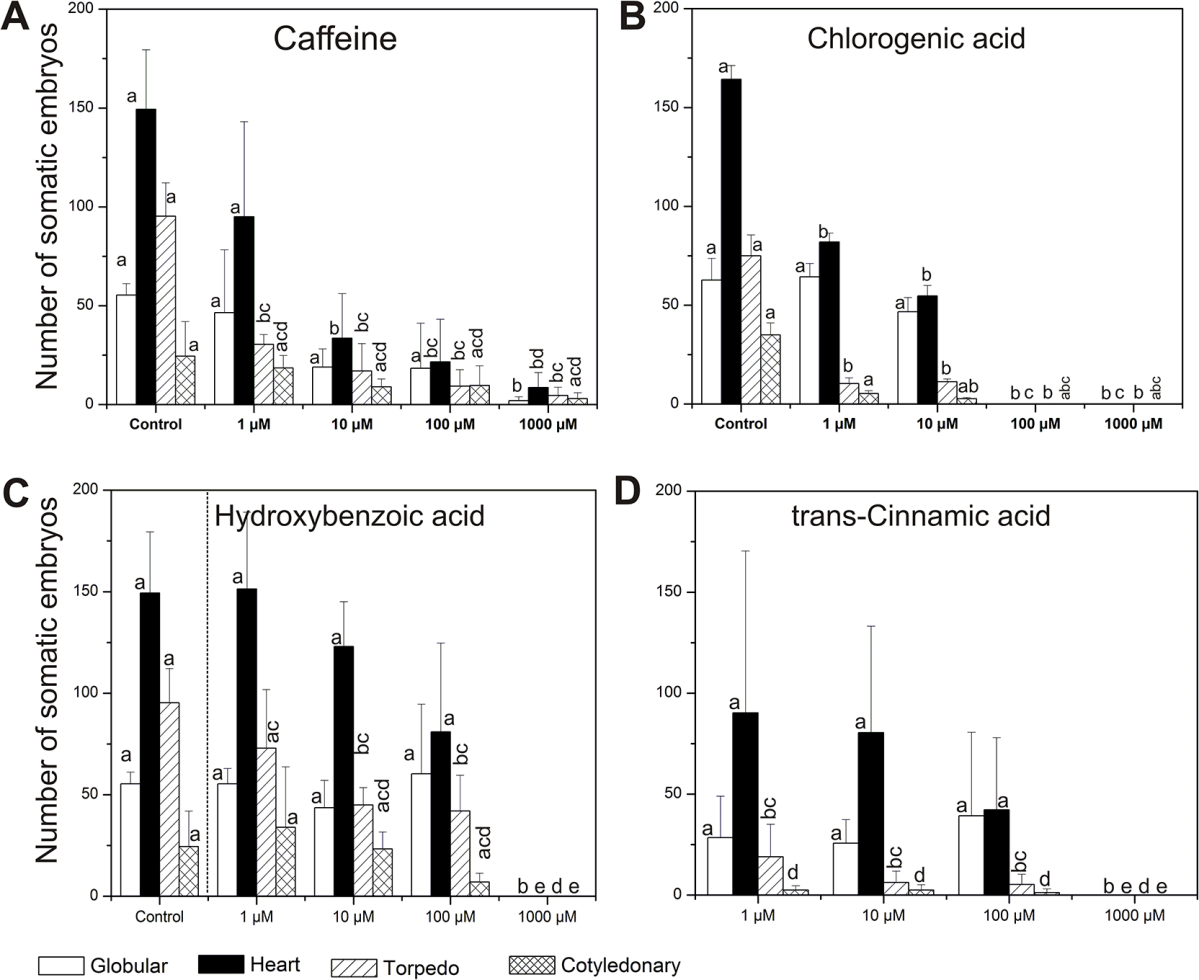
Effect of caffeine, chlorogenic acid, hydoxybenzoic acid and trans-cinnamic acid in the somatic embryogenesis of *C*. *canephora*. Explants from *C*. *canephora* leaves under embryogenic induction were supplemented with four different concentrations (1 μM, 10 μM, 100 μM and 1,000 μM) of A. Caffeine; B. Chlorogenic acid; C. Hydroxybenzoic acid, and D. Trans-cinnamic acid, at 7 dai. In contrast to the other compounds, which were diluted in the same culture medium, chlorogenic acid was diluted with dimethyl sulfoxide, which was used as a control. The number of every somatic embryo stage (globular, heart, torpedo and cotyledonary) was counted after 56 dai with and without (control) the phenolic compounds. The bars represent the mean ± SE (*n* = 3). Bars marked with different letters indicate statistically different values between each embryogenic stage at a given time according to the Tukey test (*P* ≤ 0.01). The experiments were performed three times.

We tested whether the two most abundant metabolites, caffeine and chlorogenic acid, as well as hydroxybenzoic acid and trans-cinnamic acids found in very low concentration, exert a negative response on the SE of *C*. *canephora*. Caffeine, chlorogenic acid, hydroxybenzoic acid and trans-cinnamic acid were added (Fig 1, step 4) in the induction medium of *C*. *canephora* at different concentrations (1, 10, 100 and 1000 μM; Fig 8). It was found that the addition of 1 μM of caffeine into the induction medium of *C*. *canephora* reduced the embryogenic potential by 41.3% (Fig 8A) whereas at 10 μM and 100 μM, the inhibition was 75 and 81%, respectively. Interestingly, it was observed that caffeine at the highest concentration (1,000 μM) still allowed the formation of some embryos. In the case of chlorogenic acid, its addition to the medium at concentrations of 1 and 10 μM caused a reduction in the embryogenic response of 52 and 65%, respectively, compared with the control (only with DMSO) (Fig 8B). The data show that the presence of chlorogenic acid in the induction medium strongly inhibited the transition of the H stages to T and C stages, while the same compound added at 100 and 1,000 μM caused a total SE inhibition. In addition, the presence of hydroxybenzoic acid at concentrations of 1, 10 and 100 μM moderately decreased the total number of somatic embryos (3.4, 27.6 and 41.3%, respectively) in comparison with the control (Fig 8C), whereas trans-cinnamic acid at the same concentrations has a more inhibitory effect on SE, mostly in the T and C stages (Fig 8D). However, it was also observed that both compounds added at 1,000 μM totally abolished embryo formation.

### LmmCM inhibits SE and decreases DNA methylation in *Coffea canephora*

**Fig 9:**
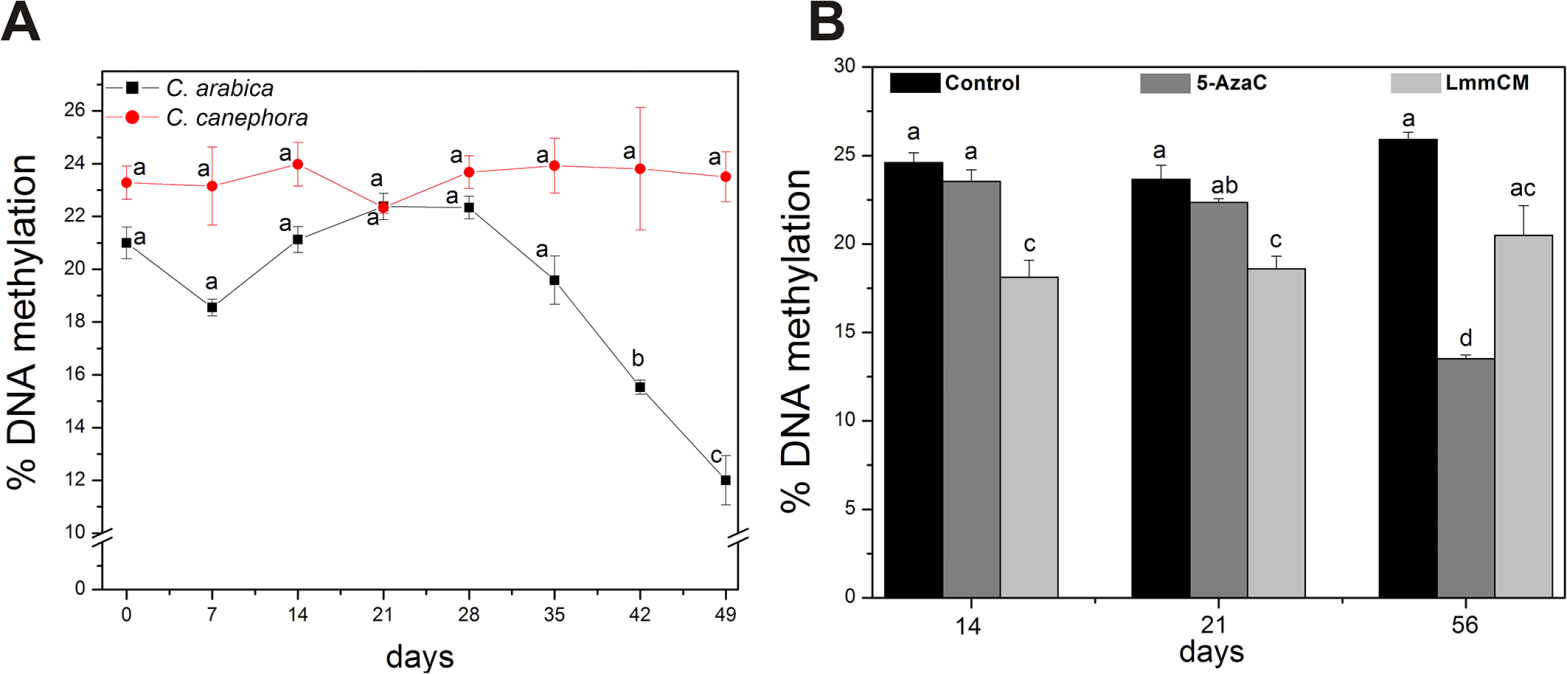
Global DNA methylation levels during the embryogenic induction of *C*. *arabica* and *C*. *canephora* and the effect of LmmCM on DNA methylation. A. Percentage of DNA methylation from explants of *C*. *arabica* and *C*. *canephora* under embryogenic induction conditions. B. Percentage of DNA methylation during the SE of *C*. *canephora* in normal conditions (control), 10 μM of 5-azacytidine (an inhibitor of DNA methylation) and with LmmCM from 7 dai *C*. *arabica*. 5-azacytidine was added every 7 days from day 7 until day 49 and the fraction of LmmCM was added only one time, at day 7 after the induction. Error bars represent the SE (*n* = 3). Bars marked with different letters indicate statistically different values between each embryogenic stage at a given time according to the Tukey test (*P* ≤ 0.01). Each experiment was carried out three times.

Recent reports have shown a correlation between DNA methylation, one of the most studied epigenetic mechanisms, and the embryogenic capacity in plants [25,31,40,41]. It has also been documented that plant-derived compounds, such as polyphenolic compounds, have the ability to act as DNA methyltransferase inhibitors [22]. In our analysis several phenolic compounds were detected from LmmCM (Fig 7, Table 1). Therefore, we wondered whether the inhibition of SE in *Coffea* by LmmCM is due to changes in DNA methylation. We analyzed global DNA methylation of both *C*. *arabica* and *C*. *canephora* explants every seven days, under embryogenic conditions, for 49 days (Fig 9A). It was observed that during the embryogenic induction process of *C*. *canephora*, 5-methyl-2´-deoxycytosine (5mdC) levels were higher than those of *C*. *arabica*, except for day 21, when both species had 22.5% DNA methylation (Fig 9A). Day 21 is the day related to the beginning of cell proliferation on the edge of the explant (Figs 3 and 4). However, unlike *C*. *canephora*, which maintains DNA methylation levels during the SE process, *C*. *arabica* presented a rapid decrease in DNA methylation levels, from 22.5% to 12% (Fig 9A). Once we indeed found changes in DNA methylation levels between *C*. *arabica* and *C*. *canephora* (Fig 9A), we assessed whether the LmmCM of *C*. *arabica* from 7 dai affects the DNA methylation in *C*. *canephora* at 14, 21 and 56 days after the induction of SE (Fig 9B). We compared the effect of the LmmCM with that of 5-AzaC at 10 μM (Fig 1, step 5). It was observed that the effect of LmmCM on DNA methylation was higher than that of 5-AzaC 10 μM at 14 and 21 dai, and this effect remained after 56 dai.

## Discussion

SE is a powerful and useful tool to understand the morphogenetic processes that occur during the development of plant embryogenesis [3]. Although the molecular changes by which somatic embryos are generated have been investigated [42–46], the mechanism by which the somatic cell transforms into the somatic embryo is still unknown. SE induction in some species seems to be more responsive to the embryogenic pathway than in others, even those from the same genus [47]. It has been proposed that the failure of SE induction in some plants is due to the secretion of compounds into the medium [15,17,18,39,48]. Therefore, we evaluated the SE response of *C*. *canephora* (a highly direct embryogenic species) in the presence of *C*. *arabica* (a deficient direct embryogenic species) explants (Fig 4), and it was observed that in the presence of *C*. *arabica*, SE in *C*. *canephora* was disrupted. This finding indicates that some substance in the surrounding medium of *C*. *arabica* affects SE in *C*. *canephora*. Some reports have even highlighted that the inhibition of SE development is produced by the accumulation of low molecular mass molecules in cultures with high cell density [14,16]. Our results show that in *C*. *arabica*, the SE-inhibitory compounds accumulate in the culture medium as early as seven dai (Fig 5), and they have a molecular mass lower than 5 kDa (Fig 6). In cultures of carrot and *L*. *leptolepis*, low molecular mass compounds such as 4-HBA, VBE and 4-PMP were identified as inhibitors of the SE response, suppressing rapid cell divisions and the development of the suspensor, respectively [15,17,18]. Therefore, we tested the 4-HBA to see whether this compound, which was not detected in any of the samples tested (S3 Fig), affects the SE of *C*. *canephora* (S4 Fig). We found that 4-HBA moderately reduced the number of embryogenic structures in *C*. *canephora* when it was added at the beginning of the induction, in comparison to the response of the LmmCM isolated from *C*. *arabica*. Unlike 4-HBA, which seems to be very specific to carrot, the LmmCM of *C*. *arabica* has a more broadly negative effect, as was demonstrated in carrot as well as coffee explants (Fig 6).

Phenolic compounds can accumulate in the culture medium, interfering with the SE process [39,49]. Therefore, from the metabolites identified by GC-MS and UPLC-ESI-ITMS (Fig 7, Table 1 and S2 Fig), we selected four compounds (caffeine, chlorogenic acid, hydroxybenzoic acid and trans-cinnamic acid), two very abundant in the LmmCM and two present in low amounts, and evaluated them in a pure form during the embryogenic response of *C*. *canephora* (Fig 8). We found that caffeine, even at the lowest concentration of 10 μM, strongly decreases the SE process in *C*. *canephora* (Fig 8A) and this compound is secreted into the culture medium, particularly in *C*. *arabica* (Fig 7). In contrast to chlorogenic acid, hydroxybenzoic acid and trans-cinnamic acid, caffeine even at the highest concentration (1,000 μM) allows somatic embryos to develop in *C*. *canephora* (Fig 8A). Similar results have also been observed during the development of zygotic embryos in coffee; these embryos have the ability to avoid caffeine autotoxicity [50]. The growth of the hypocotyl allows the root tip to move away from the caffeine-rich endosperm and facilitates the initiation of cell divisions in the root tip. On the other hand, caffeine accumulates in the cotyledons only after the cell division ends there, suggesting that the accumulation of caffeine is separated by space and time as a way to avoid autotoxicity [50]. At the molecular level, it has been found that the application of caffeine during the growth of the root meristem of *Allium cepa* induces chromosomal aberrations during cell proliferation, especially during the G2 phase and the mitotic prophase [51]. Furthermore, several studies indicate that this compound also inhibits cell plate formation during cytokinesis in plants [52–54].

It is worth noting that when chlorogenic acid was applied to *C*. *canephora* cultures, it was observed that its presence drastically decreased embryo production, up to 52% with a concentration of 1 μM (Fig 8B). This compound also interrupts embryo development beyond the H stage, whereas at concentrations ≥ 100 μM the SE process was completely inhibited. At present, the physiological role of chlorogenic acid in SE is not clear; however, it is known that high concentrations of caffeine are accompanied by a considerable accumulation of chlorogenic acid, as a pathway to form a complex to store the caffeine in the vacuole [55]. It was also observed that during coffee germination the concentration of chlorogenic acids decreases and the caffeine is released, whereas during leaf expansion an accumulation of both compounds occurs [56].

It was found that hydroxybenzoic acid at 100 μM concentration moderately inhibited SE (Fig 8C), indicating that this compound is not the responsible for the inhibition found in the SE of *C*. *canephora*. This result is consistent with previous reports, which indicate that the presence of hydroxybenzoic acid in the embryogenic cell suspension of carrot slightly decreases somatic embryo formation [48,57]. Moreover, the addition of trans-cinnamic acid to the cultures of *C*. *canephora* at the same concentration (100 μM) caused an important reduction in the formation of embryogenic structures, principally at the T and C stages, which are practically suppressed in comparison to the other treatments used (Fig 8D). Similar observations were also reported by Liu et al. [58], who found that embryos in the globular stage of *Brassica juncea* treated with trans-cinnamic acid (an auxin polar transport inhibitor) induce the fusion of cotyledons. This suggests that the correct polar transport of auxin from the early globular embryo stages is crucial for their transition to more mature embryogenic stages [59]. In addition, it has been found that hydroxycinnamic acid is accumulated principally in lines susceptible to browning, affecting SE in date palm [60]. On the other hand, the reduction of the cinnamic acid content stimulates cell division and promotes the development of early embryo structures in oak [61]. According to these results, a high concentration of trans-cinnamic acid, like that used with *C*. *canephora* (Fig 8D) could be related to the phenolic oxidation of the explants, leading to aberrations at the beginning of Pm development and preventing the generation of somatic embryos. It has been also suggested that hydroxycinnamic acid as well as the chlorogenic acid could be required to reinforce the cell wall, since these compounds, and their derivatives, are necessary to the lignification process [56,62]; probably modifying cell plasticity and interfering with the embryogenic response [60]. The results suggest that differential accumulation of phenolic compounds, in the culture medium of *C*. *arabica*, could be a major cause of the interruption in the cell division pattern affecting cellular proliferation and the SE process.

On the other hand, the regulatory mechanism by which secondary metabolites interfere with the SE process is still unknown. However, it has been reported that polyphenols, alkaloids, terpenoids and catechins, among others, can induce the loss of DNA methylation [21,22,32]. Our analysis allowed us to determine that LmmCM is composed of caffeine and chlorogenic acid, which both represent 98% of the phenolic compounds in the CM, as well catechin, epicatechin, and caffeic acid (Table 1), compounds that have been shown to have similar effects on DNA methylation [21,63,64]. Therefore, we assessed whether the LmmCM has any effect on DNA methylation and, thus, could be a mechanism by which SE is prevented in coffee (Fig 9). It was found that there is an important difference in the methylation levels between *C*. *canephora* and *C*. *arabica* (Fig 9A). *C*. *arabica* presented a 5% less DNA methylation rate than *C*. *canephora* at seven days after induction of SE. This result suggests that the inhibitory molecules present in the LmmCM could stimulate directly or indirectly the loss of DNA methylation (even more than 5-AzaC, Fig 9B), by a mechanism that needs to be further investigated.

It has been observed that caffeic acid and chlorogenic acid, two phenols isolated from coffee plants, inhibit the activity of DNA methyltransferases, particularly through a non-competitive mechanism due to an increase in the levels of *S*-adenosyl-L-homocysteine (SAH, an inhibitor of DNA methylation) [21]. Furthermore, the accumulation of SAH has also been reported during the biosynthesis of caffeine [65]. Since DNA methylation and the biosynthesis of caffeine require the same reserves of SAM, the reduction of SAM to SAH can generate a feedback inhibition of various SAM-dependent methylation processes [21]. In addition, there are reports indicating that other phenolic compounds can also inhibit DNA methyltransferase activity [21,22]. These findings suggest that the molecules present in the LmmCM, mainly phenolic compounds, could interfere with the activity of DNA methyltransferases in *C*. *arabica* and, therefore, disrupt the SE process. However it could also be possible that the decrease in DNA methylation is a consequence of the impaired SE process, with the phenolics acting upstream.

In conclusion, our results provide evidence that extracellular molecules of low molecular mass can negatively modulate the SE response and affect DNA methylation. Moreover, we showed that the release of caffeine and chlorogenic acid into the culture medium of *C*. *arabica* strongly impaired the SE process in *C*. *canephora*. Further investigations are needed to determine how these compounds affect DNA methyltransferases in this plant, and which genes are epigenetically regulated.

## Materials and Methods

### Plant materials and growth conditions

The buds used in this study were collected from plants cultivated in our research institute, Centro de Investigación Científica de Yucatán (Scientific Research Center of Yucatan), in consequence no specific permissions were required for these locations/activities. This research does not involve endangered or protected species.

Segments from buds of *C*. *arabica* derived from plants in the field were washed and disinfected with 5% Extran and 6% NaClO_3_ and rinsed with sterile distilled water. The sterile buds were cultured *in vitro* in shoot induction medium MS salts [66] supplemented with 2.96 μM thiamine-HCl, 550 μM myo-inositol, 4.06 μM nicotinic acid, 2.43 μM pyridoxine, 26.64 μM glycine, 31.6 μM cysteine, 116.85 μM sucrose, 62.5 μM BA and 0.25% (w/v) gelrite, pH 5.8 and cultured at 25 ± 2°C under a 16/8h (light/darkness) photoperiod (150 μmol m^-2^ s^-1^). Plantlets obtained from shoot induction were isolated and cultured in MS medium supplemented with 11.86 μM thiamine-HCl, 550 μM myo-inositol, 158 μM cysteine, 8.12 μM nicotinic acid, 87.64 mM sucrose and 0.25% (w/v) gelrite, pH 5.8. *C*. *canephora in vitro* plants were cultured under the same conditions as *C*. *arabica*.

*D*. *carota* seeds were sterilized with 70% ethanol and 20% NaClO_3_ for 3 min and rinsed three times with distilled sterile water and cultured *in vitro* in semisolid B_5_ medium [67] for 7 days at 25 ± 2°C under a 16/8h (light/darkness) photoperiod (150 μmol m^-2^ s^-1^). Segments 2–4 mm in length were excised from seedlings’ hypocotyls, and then two grams of tissue were transferred onto liquid B_5_ medium supplemented with 11.4 μM 2,4-dichlorophenoxyacetic acid and maintained at 100 rpm under the same conditions described above to generate cell suspension. Cell suspensions were subcultured every 14 days.

### Somatic embryogenesis induction

Plantlets of *C*. *arabica* and *C*. *canephora* were subjected under the same conditions of culture for direct embryogenic induction. Briefly, both species were pre-conditioned for 14 days in MS medium supplemented with 0.54 μM NAA and 2.32 μM KIN under the same growth conditions described above. Leaves of plantlets were cut into segments of 0.25 cm^2^, and five explants were inoculated in 50 mL liquid Yasuda modified medium contained in 250 mL flasks as previously described [37] in the presence of 5 μM BA and cultured at 25 ± 2°C in dark conditions at 55 rpm.

For embryogenic induction of *D*. *carota*, small clusters of cells were collected from seven-day-old high-density cell suspension through a stainless steel sieve of 50 μm, and then the cells were washed three times with liquid B_5_ medium without 2,4-dichlorophenoxyacetic acid and suspended in this same medium. Finally, 50 μL of packed volume cells were inoculated in 50 mL of this same medium, producing low-density cultures.

### Electron microscopy

Leaf explants of both coffee species under embryogenic induction were collected every 7 days during a temporal course of 56 days. Both sets of samples were fixed and treated as described previously [26] and analyzed with a scanning electronic microscope (GEOL JSM 6360 LV).

### Bioassays with conditioned medium of *Coffea arabica*

To evaluate the role of possible factors involved in the inhibition of SE response in *C*. *arabica*, we used the following strategies: first, the embryogenic explants of *C*. *canephora* 21 dai were co-cultured together with explants of *C*. *arabica* in the same medium seven dai (Fig 1). Second, the explants from 7, 14 and 21 dai under embryogenic conditions of *C*. *arabica* were removed and the medium (conditioned medium; CM) was used to culture embryogenic explants of *C*. *canephora* of 21 dai as shown in Fig 2B. Third, the CM from 7, 14 and 21 days of *C*. *arabica* was autoclaved and this medium was used to culture the embryogenic explants of 21 dai of *C*. *canephora*. Fourth, the CM of each flask (50 mL of medium) of *C*. *arabica* from 7, 14, 21 and 28 dai were fractionated through a membrane of cut-off 5 kDa (Amicon Ultra-15), and the obtained <5 kDa fractions of CM of *C*. *arabica* (LmmCM) were lyophilized and resuspended with 5 mL of water and sterilized by filtration with a 0.22-μm membrane. This solution was added into the culture medium of *C*. *canephora* at 21 dai and *D*. *carota* at the moment of embryogenic induction. *C*. *canephora* and *D*. *carota* cultured at induction conditions without the CM medium were used as controls and the biological response for each species was evaluated at 35 days and at 14 dai, respectively. The number of somatic embryos of *D*. *carota* was determined from three aliquots of 0.5 mL of culture for each flask.

### Analytical procedure for the metabolite profiling of LmmCM from *C*. *arabica* by UPLC-ESI-MS

Secondary metabolites were extracted from 400 mL of CM, 7 dai of SE in *C*. *arabica*. In brief, the culture medium was lyophilized until the volume reached 50 mL and then extracted three times with ethyl acetate. The extract was reduced to one mL and stored at -20°C prior to analysis. All chemicals were of analytical reagent grade (Sigma-Aldrich). HPLC-grade acetonitrile, ethanol (Fisher Scientific) and deionized water (18.2 MΩ cm, Labconco, USA) were used throughout. Formic acid, ammonium formate and phenolic compounds used as standards were Sigma reagents. Metabolite profiling was performed as described elsewhere [68], using an UltiMate 3000 liquid chromatography (Dionex, Thermo Scientific) UHPLC system equipment with a chromatographic column Luna C18 (150 x 2 mm, 3 μm) from Phenomenex and coupled with mass spectrometry detection (an ion-trap mass spectrometer AmaZon SL fitted with ESI source; Bruker Daltonics). The LC–MS system was controlled by Hystar V3.2 software, where the data were processed by Data Analysis V4.1 SP2 and QuantAnalysis V2.0 SP2 (Bruker Daltonics). The mixed standard solutions of the 34 phenolics compounds (S1 Table) containing 0.1, 0.5, 1, 5, 10 and 20 mg.L^-1^ of each compound were prepared in ethanol. All solutions were filtered, the injection volume was 10–30 μL and three replicates were always carried out. Gradient elution with two mobile phases (A—ammonium formate 10 mM + 0.2% v/v formic acid, pH 2.9; B—acetonitrile + 0.2% v/v formic acid) was as follows: 0–1 min 15% B; 1–6 min 50% B; 6–8 min 60% B; 8–11 min 60% B; 11–13 min 90% B; 14–17 min 15% B; column thermostat was set at 30°C and a total flow rate 0.2 mL min^-1^ was applied. The ESI source was operated in negative ionization mode for the compounds 1–32 and in positive ionization mode for compounds 27, 33, and 34, with the following parameters: alternate spray voltage 4,500 V; plate voltage 500 V; nebulizer gas pressure 26 psi (N_2_); dry gas 6 L min^-1^ (N_2_); source temperature 200°C and capillary exit voltage 140 V. The mass spectra were obtained by means of an UltrScan mode in the *m/z* scan range 70–400, with an ion charge control (ICC) target setting 100,000 and a maximum accumulation time 100 ms. Tuning was performed for the mixed standard solution of all 34 compounds (0.1 mg L^-1^ each) using smart parameter setting (SPS); target *m/z* 200, compound stability 100%, trap drive level 100%. Total ion chromatograms were acquired; base peak and extracted ion chromatograms were generated (*m/z* window for EIC ± 0.3 Da). For quantification, Bruker QuantAnalysis software was used, calculating the areas under the [M-H]^-1^ ions ([M+H]^+^ for positive ESI mode) from respective EIC. External calibration was carried out using a series of mixed standard solutions and the linear quadratic regression fit yielded r^2^ > 0.999 (S1 Table); on-column detection limits evaluated based on signal-to-noise ratio 3:1 were in the range 1–22 ng (10–220 μg L^-1^ as referred to the injected solution). The two samples were dissolved in 1 mL deionized water, the results presented in Table 1 correspond to the concentrations found in these solutions.

### 4-hydroxybenzyl alcohol and phenolic compounds assays on *C*. *canephora* somatic embryogenesis

Embryogenic cultures of *C*. *canephora* were treated with 4-hydroxybenzyl alcohol (Sigma, H20806) and phenolic compounds: hydroxybenzoic acid (Sigma, H6761), trans-cinnamic acid (Sigma, C6004), caffeine (Sigma, C0750) and chlorogenic acid (Sigma, C3878). Chlorogenic acid was dissolved in dimethyl sulfoxide (Sigma, D4540), while the other compounds were dissolved in the same induction medium. Then, the solutions were filter-sterilized through a membrane of 0.22 μm. 4HBA was added to the medium at the beginning of SE induction (seven dai) at concentrations of 10^–6^ M and 10^–4^ M and at 14 dai at a concentration of 10^–4^ M. Hydroxybenzoic acid, trans-cinnamic acid, caffeine and chlorogenic acid were added to the medium at seven dai at concentrations of 1, 10, 100 and 1,000 μM, and the total number of somatic embryos in every developmental stage was counted at 56 dai.

### DNA methylation

Genomic DNA extraction was carried out according to the method described by Echevarría-Machado et al. [69]. Briefly, 100 mg of leaf explants of *C*. *arabica* and *C*. *canephora* under embryogenic conditions, as shown in Fig 2, were collected every seven days from 0 to 49 days. The DNA digestion and the separation of nucleosides were performed as described in detail elsewhere [70], and the HPLC analysis was performed using the chromatographic column Kromasil C18 (250 x 4.6 mm, 5 μm from Phenomenex), and an Agilent series 1200 HPLC equipped with a pump quaternary and diode array detector. 5mdC quantification was obtained by applying the formula below to the peak areas: % 5mdC = *C* 5mdC/[*C* 5mdC + *C*dC] x 100), where *C* is the concentration of 5mdC and 2´-deoxycytosine (dC).

### Effects of LmmCM and 5-azacytidine on DNA methylation during the somatic embryogenesis of *C*. *canephora*

Embryogenic cultures of *C*. *canephora* were treated in the absence (control) or presence of 10 μM of 5-AzaC added every seven days from day 7 to day 49, whereas the addition of the LmmCM was done only one time, at day 7 after the induction. Then, the explants from the control and each treatment (5-AzaC and LmmCM) were re-collected at 14, 21 and 56 dai for DNA methylation analysis. An assessment of the global DNA methylation levels under the effects of either 5-AzaC or LmmCM was performed as described above.

### Statistical analysis

All the data were processed and analyzed using analysis of variance (ANOVA). The significance grade among the mean values was carried out using the Tukey test. Differences were considered to be significant at *P* ≤ 0.01. Data were analyzed using Origin V8 software (Data Analysis and Graphing Software).

## Supporting Information

S1 Fig Comparative effect of the LmmCM and the total molecules extracted with ethyl acetate from the conditioned medium (CM) of *Coffea arabica* on the embryogenic cultures of *Daucus carota*. A. Schematic representation of the experimental procedure. CM of *C*. *arabica* was separated and extracted as described in Materials and Methods. The two different fractions: the low molecular mass of conditioned medium (LmmCM) and the ethyl acetate phase were added separately to the embryogenic cultures of *Daucus carota*. B. Effects of LmmCM and ethyl acetate phase in the somatic embryogenesis process of *D*. *carota*.
(TIF)

S2 Fig Gas chromatography profiles of compounds extracted from the conditioned medium of 7-day-old C. arabica culture. A. Extraction of the LmmCM fraction with ethyl acetate. Peaks marked as 1, 2 and 3 correspond to the fragmentation patterns of trans-cinnamic acid (B), hydroxybenzoic acid (C) and caffeine (D), respectively.
(TIF)

S3 Fig Gas chromatography profile of the phenolic compounds extracted with ethyl acetate at day 7 from conditioned medium of *Coffea arabica*. Each peak was identified by mass spectroscopy as shown in S1 Fig. A chromatogram of a standard sample of 4-hydroxymethyl alcohol was overlapped on the chromatogram of the ethyl acetate extracted conditioned medium in order to show the retention time of this compound and its absence in the analyzed sample.
(TIF)

S4 Fig Effect of 4-hydroxybenzyl alcohol on the embryogenic cultures of *Coffea canephora*. A. *4HBA at 10^–6^ M and 10^–4^ M was added at the beginning (7 days) of the embryogenic induction of *C*. *canephora*. **4HBA at 10^–4^ M was added at 14 days after embryogenic induction of *C*. *canephora*. B. The number of embryos at different developmental stages was counted at 56 days. The control was cultivated in the absence of 4HBA. Error bars represent the SE (*n* = 3). Different letters in bars represent the statistical significance of mean differences between each embryogenic stage at a given time by the Tukey test (*P* ≤ 0.01). The experiment was carried out three times.
(TIF)

S1 Table Analytical parameters and detection limits (DL) evaluated for 34 phenolic compounds based on linear regression calibration and concentration determined in LmmCM samples from *C*. *arabica* by UPLC-ESI-ITMS. (DOCX)

## References

[1] Zimmerman JL. Somatic embryogenesis: A model for early development in higher plants. Plant Cell. 1993;5: 1411–1423. 10.1105/tpc.5.10.1411 12271037 PMC160372

[2] Loyola-Vargas VM, De-la-Peña C, Galaz-Avalos RM, Quiroz-Figueroa FR. Plant tissue culture. An intemporal set of tools In: Walker JM, Rapley R, editors. Protein and Cell Biomethods Handbook. Totowa: Humana Press; 2008 pp. 875–904. 10.1007/978-1-60327-375-6_50

[3] Vogel G. How does a single somatic cell become a whole plant? Science. 2005;309: 86 10.1126/science.309.5731.86 15994532

[4] Fuentes-Cerda CFJ, Monforte-González M, Méndez-Zeel M, Rojas-Herrera R, Loyola-Vargas VM. Modification of the embryogenic response of *Coffea arabica* by nitrogen source. Biotechnol Lett. 2001;23: 1341–1343. 10.1023/A:1010545818671

[5] Quiroz-Figueroa FR, Méndez-Zeel M, Larqué-Saavedra A, Loyola-Vargas VM. Picomolar concentrations of salycilates induce cellular growth and enhance somatic embryogenesis in *Coffea arabica* tissue culture. Plant Cell Rep. 2001;20: 679–684. 10.1007/s002990100386

[6] De-la-Peña C, Galaz-Avalos RM, Loyola-Vargas VM. Possible role of light and benzylaminopurine on biosynthesis of polyamines during the somatic embryogenesis of *Coffea canephora* . Mol Biotechnol. 2008;39: 215–224. 10.1007/s12033-008-9037-8 18228163

[7] De Vries SC, Booij H, Janssens R, Vogels R, Saris L, Loschiavo F. et al Carrot somatic embryogenesis depends on the phytohormone-controlled presence of correctly glycosylated extracellular proteins. Genes Dev. 1988;2: 462–476.

[8] De Jong AJ, Cordewener J, LoSchiavo F, Terzi M, Vandekerckhove J, Van Kammen A. et al A carrot somatic embryo mutant is rescued by chitinase. Plant Cell. 1992;4: 425–433. 10.1105/tpc.4.4.425 1498601 PMC160142

[9] Kreuger M, Van Holst GJ. Arabinogalactan proteins are essential in somatic embryogenesis of *Daucus carota* L. Planta. 1993;189: 243–248. 10.1007/BF00195083

[10] Matthys-Rochon E. Fascinating questions raised by the embryonic development in plants. Biologia. 2002;57: 1–4.

[11] De Jong AJ, Heidstra R, Spaink HP, Hartog MV, Meijer EA, Hendriks T. et al *Rhizobium* lipooligosaccharides rescue a carrot somatic embryo mutant. Plant Cell. 1993;5: 615–620. doi: 0.1105/tpc.5.6.615. 12271077 10.1105/tpc.5.6.615 PMC160299

[12] Kobayashi T, Higashi K, Kamada H. 4-Hydroxybenzyl alcohol accumulates in suspension-cell cultures and inhibits somatic embryogenesis in carrot. Physiol Plant. 2001;112: 280–284. 10.1034/j.1399-3054.2001.1120217.x 11454234

[13] Igasaki T, Akashi N, Ujino-Ihara T, Matsubayashi Y, Sakagami Y, Shinohara K. Phytosulfokine stimulates somatic embryogenesis in *Cryptomeria japonica* . Plant Cell Physiol. 2003;44: 1412–1416. 10.1093/pcp/pcg161 14701937

[14] Higashi K, Daita M, Kobayashi T, Sasaki K, Harada H, Kamada H. Inhibitory conditioning for carrot somatic embryogenesis in high-cell-density cultures. Plant Cell Rep. 1998;18: 2–6. 10.1007/s002990050522

[15] Kobayashi T, Higashi K, Sasaki K, Asami T, Yoshida S, Kamada H. Purification from conditioned medium and chemical identification of a factor that inhibits somatic embryogenesis in carrot. Plant Cell Physiol. 2000;41: 268–273. 10.1093/pcp/41.3.268 10805589

[16] Umehara M, Ogita S, Sasamoto H, Kamada H. Inhibitory factor(s) of somatic embryogenesis regulated suspensor differentiation in suspension culture of Japanese Larch (*Larix leptolepis* GORDON). Plant Biotechnol. 2004;21: 87–94. 10.5511/plantbiotechnology.21.87.

[17] Umehara M, Ogita S, Sasamoto H, Koshino H, Asami T, Fujioka S et al Identification of a novel factor, vanillyl benzyl ether, which inhibits somatic embryogenesis of Japanese larch (*Larix leptolepis* Gordon). Plant Cell Physiol. 2005;46: 445–453. 10.1093/pcp/pci041 15695457

[18] Umehara M, Ogita S, Sasamoto H, Koshino H, Nakamura T, Asami T et al Identification of a factor that complementarily inhibits somatic embryogenesis with vanillyl benzyl ether. In Vitro Cell Dev Biol-Plant. 2007;43: 203–208. 10.1007/s11627-006-9016-3 17763919

[19] Fini L, Selgrad M, Fogliano V, Graziani G, Romano M, Hotchkiss E et al Annurca apple polyphenols have potent demethylating activity and can reactivate silenced tumor suppressor genes in colorectal cancer cells. J Nutr. 2007;137: 2622–2628. 18029474 10.1093/jn/137.12.2622

[20] Fu C, Li L, Wu W, Li M, Yu X, Yu L. Assessment of genetic and epigenetic variation during long-term Taxus cell culture. Plant Cell Rep. 2012;31: 1321–1331. 10.1007/s00299-012-1251-y 22562779

[21] Lee WJ, Zhu BT. Inhibition of DNA methylation by caffeic acid and chlorogenic acid, two common catechol-containing coffee polyphenols. Carcinogenesis. 2006;27: 269–277. 10.1093/carcin/bgi206 16081510

[22] Schneider-Stock R, Ghantus A, Bajbouj K, Saikali M, Darwiche N. Epigenetic mechanisms of plant-derived anticancer drugs. Front Biosci. 2012;17: 129–173. doi: 0.2741/3919. 22201736 10.2741/3919

[23] LoSchiavo F, Pitto L, Giuliano G, Torti G, Nuti-Ronchi V, Marazziti D et al DNA methylation of embryogenic carrot cell cultures and its variations as caused by mutation, differentiation, hormones and hypomethylating drugs. Theor Appl Genet. 1989;77: 325–331. 10.1007/BF00305823 24232608

[24] Leljak-Levanic D, Bauer N, Mihaljevic S, Jelaska S. Somatic embryogenesis in pumpkin (*Cucurbita pepo* L.): Control of somatic embryo development by nitrogen compounds. J Plant Physiol. 2004;161: 229–236. 10.1078/0176-1617-01055 15022838

[25] Viejo M, Rodríguez R, Valledor L, Pérez M, Cañal M, Hasbún R. DNA methylation during sexual embryogenesis and implications on the induction of somatic embryogenesis in *Castanea sativa* Miller. Sex Plant Reprod. 2010;23: 315–323. 10.1007/s00497-010-0145-9 20552230

[26] Nic-Can GI, López-Torres A, Barredo-Pool F, Wrobel K, Loyola-Vargas VM, Rojas-Herrera R et al New insights into somatic embryogenesis: *LEAFY COTYLEDON1*, *BABY BOOM1* and *WUSCHEL-RELATED HOMEOBOX4* are epigenetically regulated in *Coffea canephora* . PLoS ONE. 2013;8: e72160 10.1371/journal.pone.0072160 23977240 PMC3748027

[27] Noceda C, Salaj T, Pérez M, Viejo M, Cañal J, Salaj J et al DNA methylation and decrease on free polyamines is associated with the embryogenic capacity of *Pinus nigra* Arn. cell culture. Trees. 2009;23: 1285–1293. 10.1007/s00468-009-0370-8

[28] Xu M, Li X, Korban S. DNA-methylation alterations and exchanges during *in vitro* cellular differentiation in rose (*Rosa hybrida* L.). Theor Appl Genet. 2004;109: 899–910. 10.1007/s00122-004-1717-6 15221146

[29] Chakrabarty D, Yu KW, Paek KY. Detection of DNA methylation changes during somatic embryogenesis of Siberian ginseng (*Eleuterococcus senticosus*). Plant Sci. 2003;165: 61–68. 10.1016/S0168-9452(03)00127-4

[30] Santos D, Fevereiro P. Loss of DNA methylation affects somatic embryogenesis in *Medicago truncatula* . Plant Cell Tiss Org Cult. 2002;70: 155–161. 10.1023/A:1016369921067

[31] Yamamoto N, Kobayashi H, Togashi T, Mori Y, Kikuchi K, Kuriyama K et al Formation of embryogenic cell clumps from carrot epidermal cells is suppressed by 5-azacytidine, a DNA methylation inhibitor. J Plant Physiol. 2005;162: 47–54. 10.1016/j.jplph.2004.05.013. 15700420

[32] Causevic A, Delaunay A, Ounnar S, Righezza M, Delmotte F, Brignolas F et al DNA methylating and demethylating treatments modify phenotype and cell wall differentiation state in sugarbeet cell lines. Plant Physiol Biochem. 2005;43: 681–691. 10.1016/j.plaphy.2005.05.011. 16046142

[33] Söndahl MR, Sharp WR. High frequency induction of somatic embryos in cultured leaf explants of *Coffea arabica* L. Z Pflanzenphysiol. 1977;81: 395–408.

[34] Yasuda T, Fujii Y, Yamaguchi T. Embryogenic callus induction from *Coffea arabica* leaf explants by benzyladenine. Plant Cell Physiol. 1985;26: 595–597.

[35] Michaux-Ferrière N, Dublin P. Embryogenése somatique chez *Coffea arabica* induction et développement des cellules embryogènes. 12è Colloque Scientifique Internationale sur le Café. Paris. 1987;418–425.

[36] Papanastasiou I, Soukouli K, Moschopoulou G, Kahia J, Kintzios S. Effect of liquid pulses with 6-benzyladenine on the induction of somatic embryogenesis from coffee (*Coffea arabica* L.) callus cultures. Plant Cell Tiss Org Cult. 2008;92: 215–225. 10.1007/s11240-007-9326-0

[37] Quiroz-Figueroa FR, Monforte-González M, Galaz-Avalos RM, Loyola-Vargas VM. Direct somatic embryogenesis in *Coffea canephora* In: Loyola-Vargas VM, Vázquez-Flota FA, editors. Plant cell culture protocols. Totowa, New Jersey: Humana Press; 2006 pp. 111–117. 10.1385/1-59259-959-1:111 16673910

[38] Matthys-Rochon E. Secreted molecules and their role in embryo formation in plants: a mini-review. Acta Biol Cracov Ser Bot. 2005;47: 23–29.

[39] Umehara M, Ikeda M, Kamada H. Endogenous factors that regulate plant embryogenesis: recent advances. Jap J Plant Science. 2007;1: 1–6.

[40] Xiao W, Custard K, Brown R, Lemmon B, Harada J, Goldbergd RB et al DNA methylation is critical for Arabidopsis embryogenesis and seed viability. Plant Cell. 2006;18: 805–814. 10.1105/tpc.105.038836 16531498 PMC1425851

[41] Levanic DL, Mihaljevic S, Jelaska S. Variations in DNA methylation in *Picea Omorika* (Panc) Purk. embryogenic tissue and the ability for embryo maturation. Prop Orn Plants. 2009;9: 3–9.

[42] Hecht V, Vielle-Calzada JP, Hartog MV, Schmidt EDL, Boutilier K, Grossniklaus U et al The Arabidopsis *SOMATIC EMBRYOGENESIS RECEPTOR KINASE 1* gene is expressed in developing ovules and embryos and enhances embryogenic competence in culture. Plant Physiol. 2001;127: 803–816. 10.1104/pp.010324 11706164 PMC129253

[43] Boutilier K, Offringa R, Sharma VK, Kieft H, Ouellet T, Zhang L et al Ectopic expression of BABY BOOM triggers a conversion from vegetative to embryonic growth. Plant Cell. 2002;14: 1737–1749. 10.1105/tpc.001941 12172019 PMC151462

[44] Rojas-Herrera R, Quiroz-Figueroa FR, Sánchez-Teyer F, Loyola-Vargas VM. Molecular analysis of somatic embryogenesis: An overview. Physiol Mol Biol Plants. 2002;8: 171–184.

[45] Ikeda M, Umehara M, Kamada H Embryogenesis-related genes; Its expression and roles during somatic and zygotic embryogenesis in carrot and Arabidopsis. Plant Biotechnol. 2006;23: 153–161. 10.5511/plantbiotechnology.23.153.

[46] Yang X, Zhang X. Regulation of somatic embryogenesis in higher plants. Crit Rev Plant Sci. 2010;29: 36–57. 10.1080/07352680903436291

[47] Fehér A. The initiation phase of somatic embryogenesis: what we know and what we don´t. Acta Biol Sczeg. 2008;52: 53–56.

[48] Kobayashi T, Higashi K, Kamada H Stimulatory and inhibitory conditioning factors that regulate cell proliferation and morphogenesis in plant cell cultures. Plant Biotechnol. 2001;18: 93–99. 10.5511/plantbiotechnology.18.93.

[49] Kouakou TH, Waffo-Téguo P, Kouadio YJ, VAlls J, Richard T, Decendit A et al Phenolic compounds and somatic embryogenesis in cotton (*Gossypium hirsutum* L.). Plant Cell Tiss Org Cult. 2007;90: 25–29. 10.1007/s11240-007-9243-2

[50] Friedman J, Waller G. Caffeine hazards and their prevention in germinates seed of coffee (*Coffea arabica* L.). J Chem Ecol. 1983;9: 1099–1106. 10.1007/BF00982214 24407803

[51] Hernández P, Mingo R, González A, López-Sáez J Relationship of chromosomal damage induced by caffeine to growth temperature and ATP level in proliferating cells. Mut Res. 1986;164: 327–333. 10.1016/0165-1161(86)90003-8. 3773927

[52] Verma DPS. Cytokinesis and building of the cell plate in plants. Annu Rev Plant Physiol Plant Mol Biol. 2001;52: 751–784. 10.1146/annurev.arplant.52.1.751 11337415

[53] Valster AH, Hepler PK. Caffeine inhibition of cytokinesis: effect on the phrangmoplast cytoskeleton in living *Tradescantia* stamen hair cells. Protoplasma. 1997;196: 155–166. 10.1007/BF01279564

[54] Hepler PK, Bonsignore C. Caffeine inhibition of cytokinesis: ultrastructure of cell plate formation/degradation. Protoplasma. 1990;157: 182–192. 10.1007/BF01322651

[55] Mösli WS, Baumann TW. Compartmentation of caffeine and related purine alkaloids depends exclusively on the physical chemistry of their vacuolar complex formation with chlorogenic acids. Phytochemistry. 1996;42: 985–996. 10.1016/0031-9422(96)00072-6

[56] Aerts RJ, Baumann TW. Distribution and utilization of chlorogenic acid in *Coffea* seedlings. J Exp Bot. 1994;45: 497–503. doi: 1994_JEB_497_26490.

[57] Fridborg G, Pedersen M, Landstrom L-E, Eriksson T. The effect of activated charcoal on tissue cultures; adsorption of metabolites inhibiting morphogenesis. Physiol Plant. 1978;43: 104–106. 10.1111/j.1399-3054.1978.tb01575.x

[58] Liu C, Xu Z-H, Chua N-H. Auxin polar transport is essential for the establishment of bilateral symmetry during early plant embryogenesis. Plant Cell. 1993;5: 621–630. 10.1105/tpc.5.6.621 12271078 PMC160300

[59] Petrásek J, Friml J. Auxin transport routes in plant development. Development. 2009;136: 2675–2688. 10.1242/10.1242/dev.030353 19633168

[60] Mounir EB, Ismail EH. Characterization of two non constitutive hydroxycinnamic acid derivatives in Date Palm (*Phoenix dactylifera* L.) callus in relation with tissue browning. Biotechnology. 2004;3: 155–159. 15292581

[61] Cvikrová M, Malá J, Hrubcová M, Eder J, Zón J, Macháčková I. Effect of inhibition of biosynthesis of phenylpropanoids on sessile oak somatic embryogenesis. Plant Physiol Biochem. 2003;41: 251–259. 10.1016/S0981-9428(03)00016-0.

[62] Santiago R, de Armas R, Fontaniella B, Vicente C, Legaz M. Changes in soluble and cell wall-bound hydroxycinnamic and hydroxybenzoic acids in sugarcane cultivars inoculated with *Sporisorium scitamineum* sporidia. Eur J Plant Plathol. 2009;124: 439–450. 10.1007/s10658-009-9431-5

[63] Barrès R, Yan J, Egan B, Treebak JT, Rasmussen M, Fritz T et al Acute exercise remodels promoter methylation in human skeletal muscle. Cell Metab. 2012;15: 405–411. 10.1016/j.cmet.2012.01.001 22405075

[64] Crescenti A, Solà R, Valls RM, Caimari A, del Bas JM, Anglés N et al Cocoa consumption alters the global DNA methylation of peripheral leukocytes in humans with cardiovascular disease risk factors: A randomized controlled trial. PLoS ONE. 2013;8: e65744 10.1371/journal.pone.0065744 23840361 PMC3694105

[65] Koshiishi C, Kato A, Yama S, Crozier A, Ashihara H. A new caffeine biosynthetic pathway in tea leaves: utilisation of adenosine released from the S-adenosyl-L-methionine cycle. FEBS Lett. 2001;499: 50–54. 11418110 10.1016/s0014-5793(01)02512-1

[66] Murashige T, Skoog F A. revised medium for rapid growth and bioassays with tobacco tissue cultures. Physiol Plant. 1962;15: 473–497. 10.1111/j.1399-3054.1962.tb08052.x

[67] Gamborg OL, Miller RA, Ojima K. Nutrient requirements of suspension cultures of soybean root cells. Exp Cell Res. 1968;50: 151–158. 10.1016/0014-4827(68)90403-5 5650857

[68] Alcázar Magana A, Wrobel K, Torres Elguera JC, Corrales Escobosa AR, Wrobel K. Determination of small phenolic compounds in tequila by liquid chromatography with ion trap mass spectrometry detection. Food Anal Method. 2015;8: 864–872. 10.1007/s12161-014-9967-7

[69] Echevarría-Machado I, Sánchez-Cach L, Hernández-Zepeda C, Rivera-Madrid R, Moreno-Valenzuela O. A simple and efficient method for isolation of DNA in high mucilaginous plant tissues. Mol Biotechnol. 2005;31: 129–135. 10.1385/MB:31:2:129 16170213

[70] De-la-Peña C, Nic-Can G, Ojeda G, Herrera-Herrera J, Lopez-Torres A, Wrobel K et al KNOX1 is expressed and epigenetically regulated during *in vitro* conditions in *Agave* spp. BMC Plant Biology. 2012;12: 203 10.1186/1471-2229-12-203 23126409 PMC3541254

