# Supplementary figures and images for: Somatic Embryogenesis: Identified Factors that Lead to Embryogenic Repression. A Case of Species of the Same Genus
Source: PLoS One. 2015 Jun 3;10(6):e0126414. doi: 10.1371/journal.pone.0126414 (PMC4454440; doi:10.1371/journal.pone.0126414)

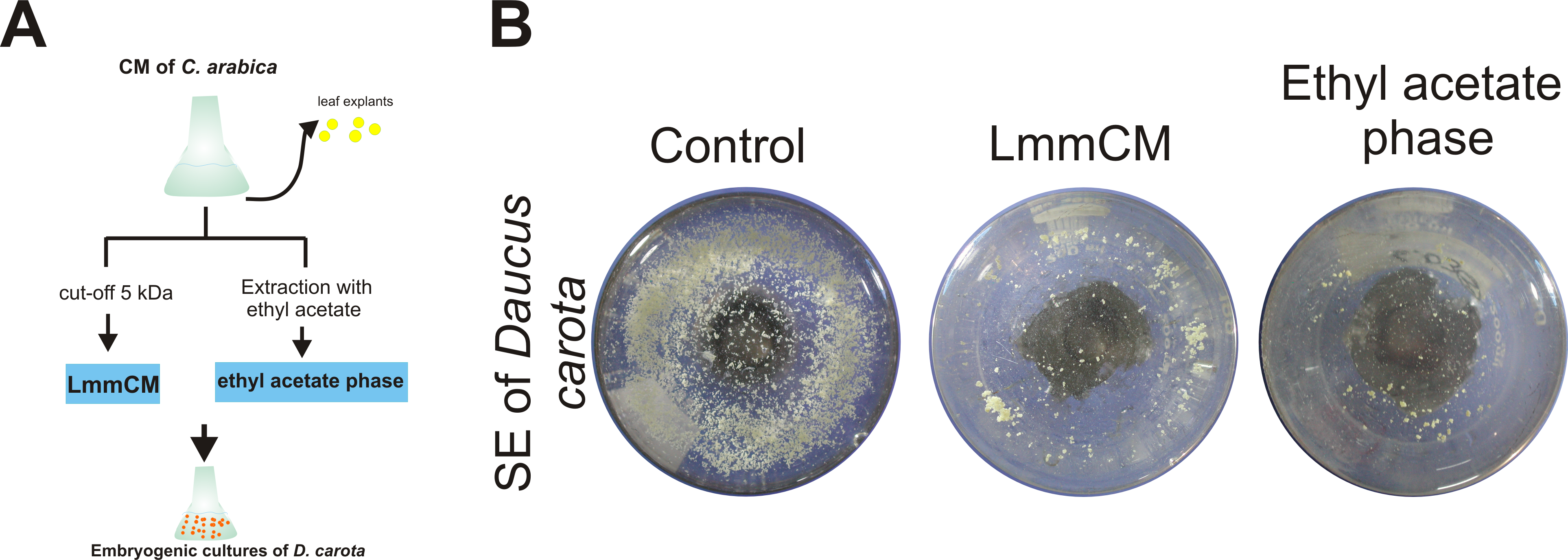

Supplement: S1 Fig — A. Schematic representation of the experimental procedure. CM of C. arabica was separated and extracted as described in Materials and Methods. The two different fractions: the low molecular mass of conditioned medium (LmmCM) and the ethyl acetate phase were added separately to the embryogenic cultures of Daucus carota. B. Effects of LmmCM and ethyl acetate phase in the somatic embryogenesis process of D. carota. (TIF) [file pone.0126414.s001.tif]

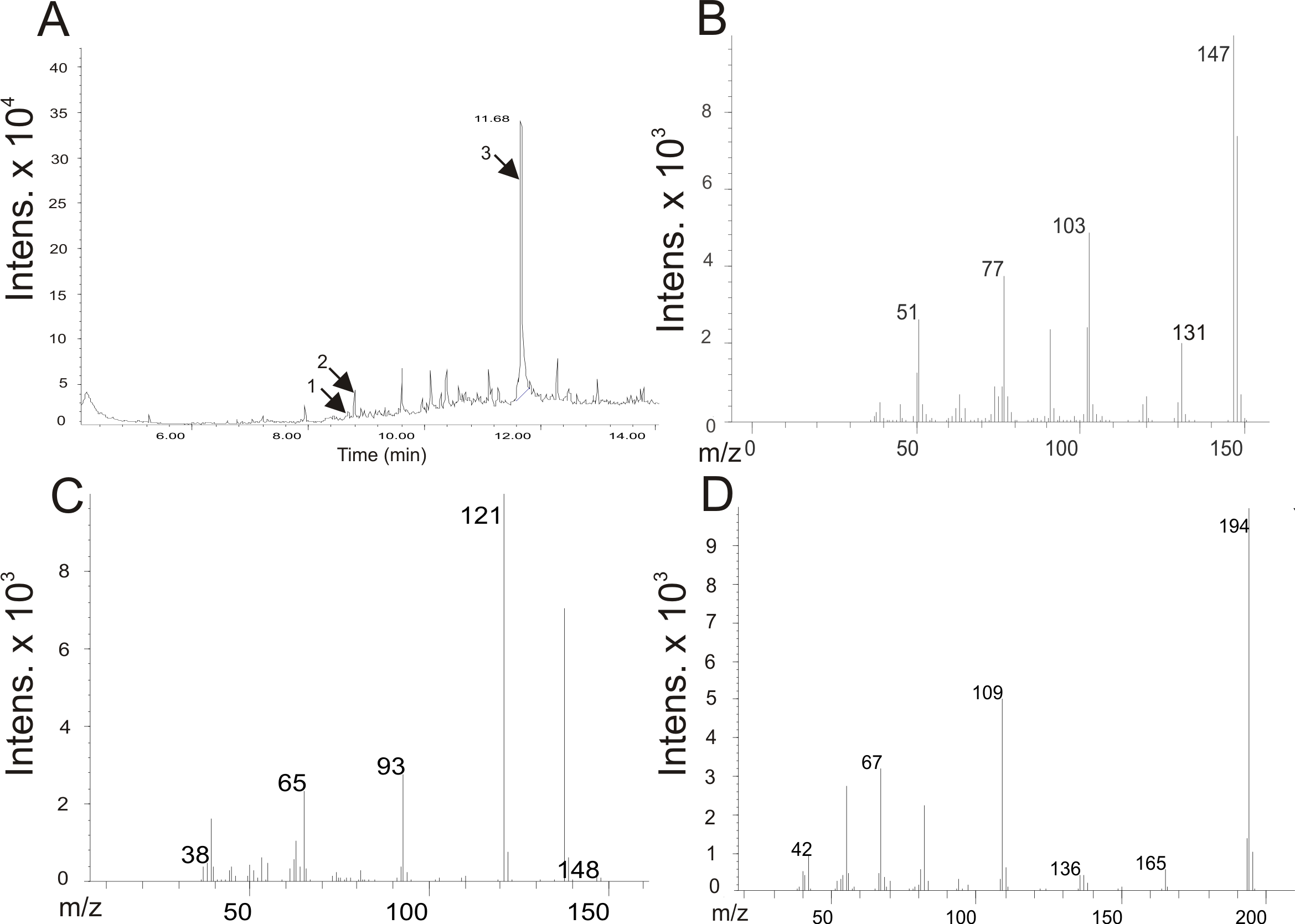

Supplement: S2 Fig — A. Extraction of the LmmCM fraction with ethyl acetate. Peaks marked as 1, 2 and 3 correspond to the fragmentation patterns of trans-cinnamic acid (B), hydroxybenzoic acid (C) and caffeine (D), respectively. (TIF) [file pone.0126414.s002.tif]

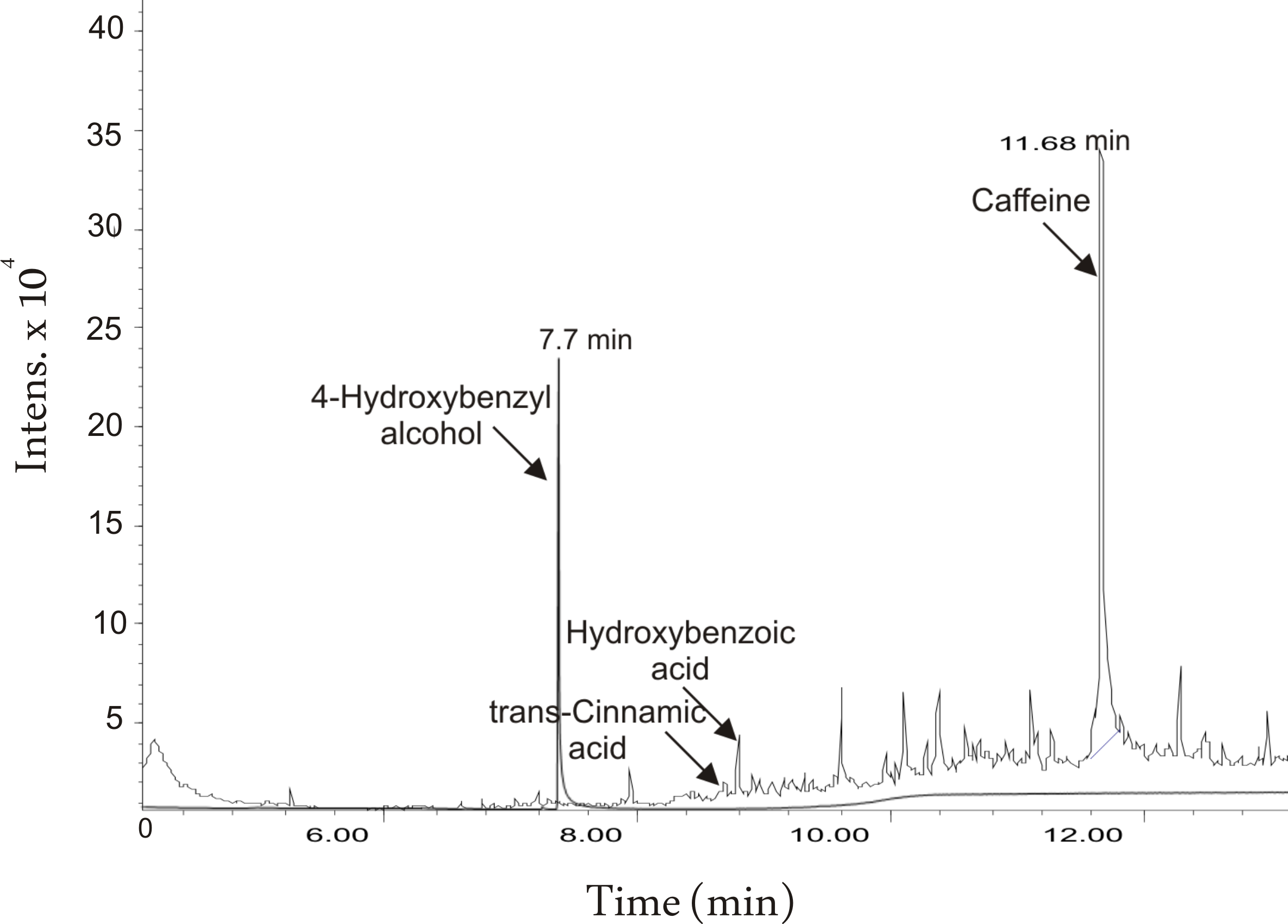

Supplement: S3 Fig — Each peak was identified by mass spectroscopy as shown in S1 Fig. A chromatogram of a standard sample of 4-hydroxymethyl alcohol was overlapped on the chromatogram of the ethyl acetate extracted conditioned medium in order to show the retention time of this compound and its absence in the analyzed sample. (TIF) [file pone.0126414.s003.tif]

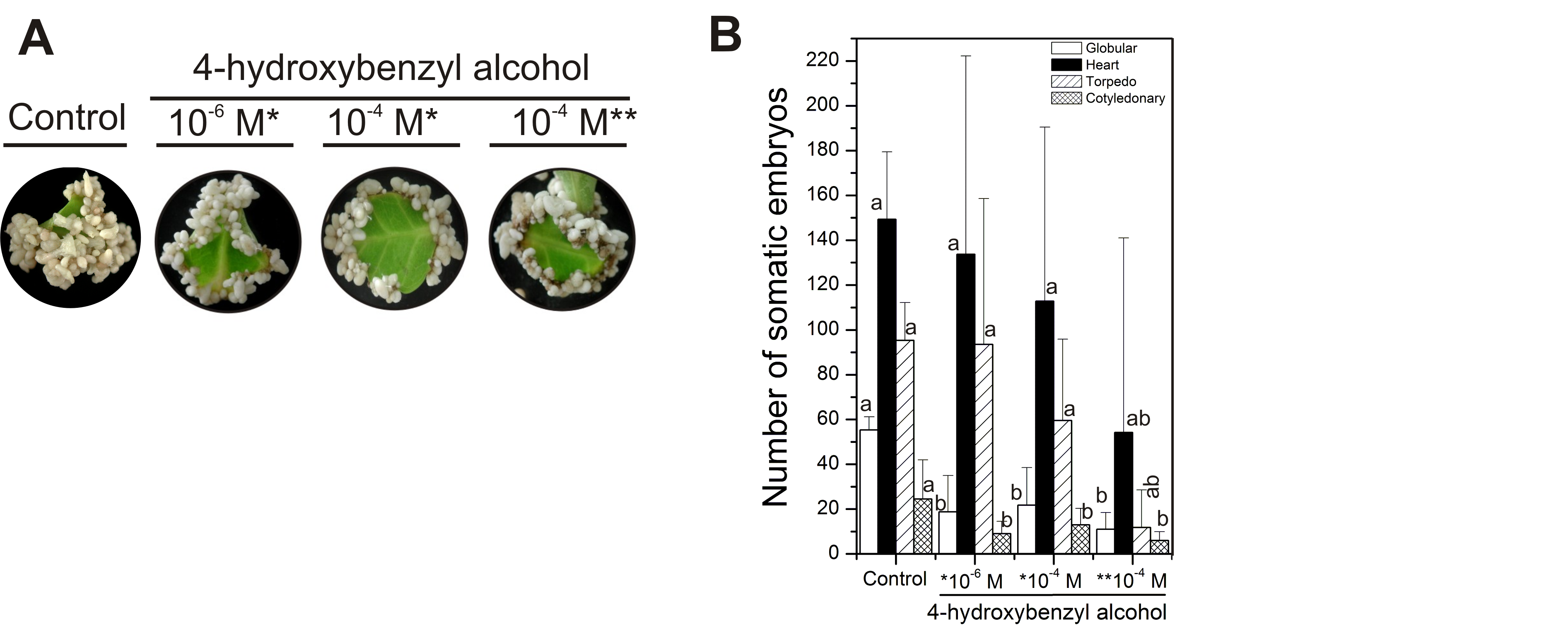

Supplement: S4 Fig — A. *4HBA at 10–6 M and 10–4 M was added at the beginning (7 days) of the embryogenic induction of C. canephora. **4HBA at 10–4 M was added at 14 days after embryogenic induction of C. canephora. B. The number of embryos at different developmental stages was counted at 56 days. The control was cultivated in the absence of 4HBA. Error bars represent the SE (n = 3). Different letters in bars represent the statistical significance of mean differences between each embryogenic stage at a given time by the Tukey test (P ≤ 0.01). The experiment was carried out three times. (TIF) [file pone.0126414.s004.TIF]
