# Supplementary material for: Somatic Embryogenesis: Identified Factors that Lead to Embryogenic Repression. A Case of Species of the Same Genus
Source: PLoS One. 2015 Jun 3;10(6):e0126414. doi: 10.1371/journal.pone.0126414 (PMC4454440; doi:10.1371/journal.pone.0126414)
Supplement: S1 Table — (DOCX) [file pone.0126414.s005.docx]

**Table S1** Analytical parameters and detection limits (DL) evaluated for 34 phenolic compounds based on linear regression calibration and concentration determined in LmmCM samples from *C. arabica* by UPLC-ESI-ITMS.

| Phenolic compound | | Linear regression | R^2^ | ^a^DL  (µg.L^-1^) | ^b^Concentration ± SD (µg.L^-1^) | |
| --- | --- | --- | --- | --- | --- | --- |
| Negative ESI | |  |  |  | **LmmCM_1_** | **LmmCM_2_** |
| 1 | Gallic acid | A = -27283.9 c^2 + 1765301.8 c + 179002.6 | 0.99979 | 2.35 | n/d | n/d |
| 2 | Homogentisic acid | A = -14645.8 c^2 + 1816237.2 c -233561.9 | 0.99976 | 2.66 | n/d | n/d |
| 3 | Protocatechuic acid | A = 1142178.8 c **-**4719.3 | 1.00000 | 2.56 | n/d | n/d |
| 4 | 3,4-Dihydroxyphenyl-  acetic acid | A = 8277.3 *c^*2 + 499117.8 c -114897.3 | 0.99986 | 11.12 | n/d | n/d |
| 5 | Chlorogenic acid | A = 3711744.9 c + 959271.1 | 0.99914 | 2.57 | 2430±20 | 5630±20 |
| 6 | Catechin | A = -52723.8 c^2 + 5185403.8c + 221578.8 | 0.99997 | 2.80 | 40±1 | 203±1 |
| 7 | Resorcinol | A = -1264.5 c^2 + 107837.4 c -3947.3 | 1.00000 | 66.96 | n/d | n/d |
| 8 | 2,5-Dihydroxybenzoic  acid | A = 1703501.0 c -93884.3 | 0.99971 | 7.79 | n/d | n/d |
| 9 | 4-Hydroxybenzoic  acid | A=308760.4 c + 65975.7 | .99970 | 10.91 | n/d | n/d |
| 10 | Epicatechin | A = -37724.6 c^2 + 2866499.0 c + 367154.9 | 0.99971 | 3.22 | 34±2 | 117±2 |
| 11 | 2,3-Dihydroxybenzoic  acid | A = -34976.7 c^2 + 2567671.5 c + 345695.9 | 0.99970 | 5.83 | n/c | n/c |
| 12 | Caffeic acid | A = -29836.4 c^2 + 3511871.2 c + 138126.5 | 1.00000 | 3.00 | 12.6±1 | 57.4±6 |
| 13 | Catechol | A = -2965.1 c^2 + 369842.4 c + 83884.4 | 0.99926 | 17.90 | n/d | n/d |
| 14 | Vanillic acid | A = -1257.7 c^2 + 200503.1 c + 7294.1 | 0.99998 | 19.06 | n/d | n/d |
| 15 | Syringic acid | A = -2788.9 c^2 + 542130.9 c + 26899.9 | 1.00000 | 10.04 | n/d | n/d |
| 16 | 2,4-Dihydroxybenzoic  acid | A = 2082433.6 c -76759.2 | 0.99998 | 1.96 | n/d | n/d |
| 17 | Homovanillic acid | A = 847717.4 c + 18531.4 | 0.99952 | 8.17 | n/d | n/d |
| 18 | 3-Hydroxybenzoic  acid | A = 528352.2 c -102156.8 | 0.99982 | 9.65 | n/d | n/d |
| 19 | 4-Hydroxyphenyl-propionic acid | A = 1585788.1c -226651.0 | 0.99930 | 2.85 | n/d | n/d |
| 20 | 3,4-Dihydroxyphenyl-propionic acid | A = -5199.4 c^2 + 441399.8 c + 40767.9 | 0.99985 | 10.32 | n/d | n/d |
| 21 | 2,6-Dihydroxybenzoic  acid | A = 3770099.2 c + 763373.4 | 0.99989 | 2.34 | n/d | n/d |
| 22 | p-Coumaric acid | A = 1389312.3 c + 286245.0 | 0.99966 | 4.86 | n/d | n/d |
| 23 | Synapic acid | A = -29312.0 c^2 + 3218654.8 c -131613.5 | 0.99997 | 2.34 | n/d | n/d |
| 24 | Ferulic acid | A = 2288514.1 c -281345.6 | 0.99970 | 1.81 | 13.9±1 | 38.7±3 |
| 25 | 3,4-Dimethoxybenzoic  acid | A = 198435.6 c + 13488.1 | 0.99995 | 25.64 | n/d | n/d |
| 26 | m-Coumaric acid | A = 3202812.3 c -175737.6 | 0.99991 | 1.28 | n/d | n/d |
| 27 | o-Anisic acid | A = 37200.7 c -1613.1 | 0.99953 | 75.00 | n/d | n/d |
| 28 | trans 2-Hydroxy-cinnamic acid | A = 13254.6 c^2 + 1431783.9 c -148330.3 | 0.99993 | 2.67 | n/d | n/d |
| 29 | Salicylic acid | A = -15144.6 c^2 + 1415660.7 c + 184253.4 | 0.99987 | 9.24 | 103±13 | 304±30 |
| 30 | Benzoic acid | A = 31316.8 c + 8467.8 | 0.99945 | 56.39 | 127±5 | 384±29 |
| 31 | Cinnamic acid | A = 183553.74 c + 18898.1 | 0.99997 | 27.99 | n/d | n/d |
| 32 | 4-Methoxycinnamic  acid | A = 8764.1 c^2 + 51311.4 c + 12265.3 | 1.00000 | 24.92 | n/d | n/d |
| Positive ESI | |  |  |  |  |  |
| 27 | o-Anisic acid | A = -549347.2 c^2 + 29698308.2 c + 4659249.8 | 0.99926 | 4.29 | n/d | n/d |
| 33 | Caffeine | A = -952624.7 c^2 + 59372240.1 c + 8283206.7 | 0.99950 | 5.54 | 8574±57 | 17045±68 |
| 34 | Coumarin | A = 23993432.2 c + 4553923.3 | 0.99901 | 27.99 | n/d | n/d |

^a^ Calibration detection limit evaluated as signal-to-noise ratio 3:1

^b^ Standard deviation evaluated based on three successive injections of the LmmCM samples
